# Identification and characterization of ACR gene family in maize for salt stress tolerance

**DOI:** 10.3389/fpls.2024.1381056

**Published:** 2024-04-30

**Authors:** Hui Fang, Tingyu Shan, Haijing Gu, Junyu Chen, Yingxiao Qi, Yexiong Li, Muhammad Saeed, Jinchao Yuan, Ping Li, Baohua Wang

**Affiliations:** ^1^ Ministry of Agricultural Scientific Observing and Experimental Station of Maize in Plain Area of Southern Region, School of Life Sciences, Nantong University, Nantong, Jiangsu, China; ^2^ Department of Agricultural Sciences, Government College University, Faisalabad, Pakistan; ^3^ Qidong Ruichao Farm, Nantong, Jiangsu, China

**Keywords:** *ACR* gene family, *Zea mays*, expression patterns, salt stress, ectopic overexpression

## Abstract

**Background:**

Members of the ACR gene family are commonly involved in various physiological processes, including amino acid metabolism and stress responses. In recent decades, significant progress has been made in the study of ACR genes in plants. However, little is known about their characteristics and function in maize.

**Methods:**

In this study, ACR genes were identified from the maize genome, and their molecular characteristics, gene structure, gene evolution, gene collinearity analysis, cis-acting elements were analyzed. qRT-PCR technology was used to verify the expression patterns of the ZmACR gene family in different tissues under salt stress. In addition, Ectopic expression technique of ZmACR5 in Arabidopsis thaliana was utilized to identify its role in response to salt stress.

**Results:**

A total of 28 ZmACR genes were identified, and their molecular characteristics were extensively described. Two gene pairs arising from segmented replication events were detected in maize, and 18 collinear gene pairs were detected between maize and 3 other species. Through phylogenetic analysis, three subgroups were revealed, demonstrating distinct divergence between monocotyledonous and dicotyledonous plants. Analysis of ZmACR cis-acting elements revealed the optional involvement of ZmACR genes in light response, hormone response and stress resistance. Expression analysis of 8 ZmACR genes under salt treatment clearly revealed their role in the response to salt stress. Ectopic overexpression of ZmACR5 in Arabidopsis notably reduced salt tolerance compared to that of the wild type under salt treatment, suggesting that ZmACR5 has a negative role in the response to salt stress.

**Conclusion:**

Taken together, these findings confirmed the involvement of ZmACR genes in regulating salt stress and contributed significantly to our understanding of the molecular function of ACR genes in maize, facilitating further research in this field.

## Introduction

The ACT domain, commonly referred to as an amino acid-binding domain, was first found in 1995 when the crystal structure of D-3-phosphoglycerate dehydrogenase in *E. coli* (3PGDH) was resolved (Schuller et al., 1995). This domain consists of four subunits, each containing one ACT domain. The ACT domain typically contains four β strands and two α helices arranged in a βαββαβ structure (Grant, 2006; Lang et al., 2014). Through the use of position-specific iterating-Blast (PSI-Blast), the ACT domain has been found in a wide variety of proteins (Aravind and Koonin, 1999). The specific domain was subsequently designated the ACT domain after the initial letters of three bacterial proteins, aspartate kinase, chorismate mutase and tyrA (prephenate dehydrogenase) (Liberles et al., 2005). The ACT domain functions as a regulatory ligand-binding domain and has a common ligand-binding mechanism, making it a component of diverse proteins during protein evolution (Aravind and Koonin, 1999; Anantharaman et al., 2001; Chipman and Shaanan, 2001). Typically consisting of 70-90 amino acids, the ACT domain is often located at the C-terminus of proteins and serves as the binding site for its allosteric regulator of protein function (Chipman and Shaanan, 2001; Grant, 2006).

Proteins containing ACT domains are well known to interact with amino acids and directly or indirectly regulate amino acid metabolism (Aravind and Koonin, 1999; Chipman and Shaanan, 2001; Ettema et al., 2002; Grant, 2006; Okamura and Hirai, 2017). The ACT domain is encoded by metabolic enzymes, such as aspartate kinase/homoserine dehydrogenase (Marina et al., 2004; Dumas et al., 2012), prephenate dehydratase (Hsu et al., 2004), phenylalanine hydroxylase (Pey et al., 2004), and aspartokinase (Tunca et al., 2004). In addition, several transcription factors that have an ACT domain have been found (Wilson et al., 1995; Schreiter et al., 2003; Devedjiev et al., 2004; Feller et al., 2006). The genes that code for the ACT domain can be divided into a group called the ACR genes, which contain 2-4 ACT domain repeats. Several ACR genes have been identified and characterized.

In *Arabidopsis*, 12 members of the ACR gene family, namely, *ACR1*-*ACR12*, which contain 2-4 ACT domain repeats, have been identified (Hsieh and Goodman, 2002; Sung et al., 2011). Among them, *ACR4*, a putative receptor kinase, was further analyzed and found to be expressed in the outer cellular layers of embryos and plants, indicating that it may be related to embryogenesis (Tanaka et al., 2002). Moreover, the *Arabidopsis* ACR11 protein may function as a regulatory protein that is involved in glutamine metabolism or signaling in the chloroplast (Sung et al., 2011; Takabayashi et al., 2016), as well as nitrogen assimilation (Osanai et al., 2017). A recent study showed that the *Arabidopsis ACR11* gene might also play a role in regulating salicylic acid (SA)-related defense mechanisms by controlling the accumulation of reactive oxygen species (ROS) and SA (Singh et al., 2018). In rice, 9 ACR genes containing 4 copies of the ACT domain were identified (Liu, 2006). Phylogenetic analysis suggested that the *Arabidopsis* and rice ACR proteins may have evolved from an ancient common ancestor. The rice *ACR7* gene was further shown to bind Gln and act as a Gln sensor in rice leaves (Hayakawa et al., 2006). Additionally, the nuclear protein OsACR9 was proved to act as a Gln sensor through its spatiotemporal expression compared to that of Gln-responsive N assimilatory genes in rice (Kudo et al., 2008).

Maize, one of the 3 largest grain crops in the world, plays an irreplaceable role in global food security and nutrition. Maize has a wide range of uses and is also an important source of biofuels in addition to being utilized for human and animal feed (Ranum et al., 2014). However, little is known about the characteristics and function of the ACR gene family in maize. In this study, we carried out a genome-wide identification and systematic analysis of the ACR gene family in maize based on B73 genome sequences (Bukowski et al., 2018). This study aimed to shed light on the functions of *ZmACR* members and to facilitate further comprehensive research on ACR genes in maize.

## Materials and methods

### Identification and characterization of ACR gene family members in maize

To identify the ACR gene sequences in maize, we used the Ensembl website (http://plants.ensembl.org/index.html) to download the B73 genome database, which included whole-genome files and gene annotation files (gff3). Based on the ACR domain (PF01842) in Pfam (http://pfam.xfam.org/), we employed a hidden Markov model (HMM) to query the ACR protein sequences throughout the entire B73 genome by using default parameters in HMMER software. The ZmACR proteins were identified using the Hummsearch program in the Linux system 5.2.38 (Jeanmougin et al., 1998). Subsequently, the Pfam, SMART (Simple Modular Architecture Research Tool, http://smart.embl-heidelberg.de) and Conserved domain (CDD, http://www.NCBI.nlm.NIH.gov/Structure/cdd/wrpsb) were utilized to confirm and eliminate redundant sequences. Subsequently, ExPASy Proteomics Server software (http://www.expasy.org) was used to predict the molecular weights (MWs), isoelectric points (PIs), and amino acid lengths of all identified ACR genes in maize.

### Phylogenetic tree construction and conserved protein motif analysis of the ACR genes in maize

A phylogenetic tree was constructed using the Maximum Composite Likelihood (MCL) model of neighbor-joining (NJ) algorithm in MEGA7 software, and 1,000 iterations of bootstrapping were performed. The Evolview (http://www.omicsclass.com/article/671) website was browsed to further modify and enrich the evolutionary tree. Motif analysis based on ZmACR protein sequences was carried out using MEME Suite 5.3.3, and the detailed information of the motif was subsequently viewed through the MEME website (http://meme-suite.org/). Finally, TBtools software was used to edit and save the retrieved images (Chen et al., 2020).

### Gene structure and *cis*-acting element analysis of the ACR genes in maize

The Gene Structure Display Server (GSDS, http://gsds.gao-lab.org/) was used to analyze the gene structures of the ACR family genes. To explore the regulation of gene expression, the 1.5 kb sequence upstream of the initiation codon of each *ZmACR* gene was extracted to analyze the *cis*-acting elements of these genes. The Plant CARE website (*Cis*-acting Regulatory Element, http://bioinformatics.psb.ugent.be/webtools/plantcare/html/) was used to further analyze the *cis*-acting elements according to the obtained 1.5 kb sequences, and the obtained *cis*-acting element information was subsequently mapped through the GSDS website. The distribution of *cis*-acting elements within the promoters was visualized with TBtools (Chen et al., 2020).

### Chromosomal location, Ka/Ks analysis and collinearity analysis

The chromosomal localization of *ZmACR* genes was determined from whole-genome files and gene annotation files, and the repeat pattern of each *ZmACR* gene was analyzed via BLAST. Then, we used MapChart and Adobe Illustrator CS6 software to construct a map of the chromosomes. *Ka/Ks* represents the ratio between the nonsynonymous substitution rate (*Ka*) and the synonymous substitution rate (*Ks*) of two protein-coding genes. After alignment, the synonymous substitution rate (*Ks*) and nonsynonymous substitution rate (*Ka*) of nucleotides in *ZmACR* family genes, as well as their ratios, were calculated by the KaKs_Calculator2.0 tool. The threshold for *P*-value of KA, KS and their ratio was set to 1×10^-10^. MCScan toolkit (V1.1) (Wang et al., 2012; VanBuren et al., 2018) implemented in python (https://github.com/tanghaibao/jcvi/wiki/MCscan-(Python-version)) was used to analyze the collinearity of the *ZmACR* genes. The collinear genes were identified in the whole genome based on the sequence alignment of proteins. Finally, the results were visualized using Circos (http://circos.ca/) software.

### Secondary structure prediction and three-dimensional model construction of ZmACR proteins

The secondary structure of ZmACR proteins was predicted using the online website Prabi (https://npsa-prabi.ibcp.fr). In order to delve deeper into the protein structure of the ZmACR proteins, their tertiary structures were predicted based on their protein sequences. Subsequently, the online tools SWISS-MODEL (https://swissmodel.expasy.org/) was amployed to generate the 3D models of ZmACR proteins through homologous protein modeling method (Waterhouse et al., 2018).

### Subcellular localization analysis of the ZmACR proteins

We utilized the CELLO online website (http://cello.life.nctu.edu.tw/) to predict the subcellular localization of the ZmACR genes based on their protein sequences (Yu et al., 2006). By submitting the protein sequences to the website, we obtained potential organelle locations. The results were then visualized using TBtools. To verify the accuracy of the bioinformatic prediction results, we randomly selected 4 *ZmACR* genes and investigated their expression localization. We amplified the full-length CDSs of the genes using gene-specific primers (**Supplementary Table S1**) and KOD One TM PCR Master Mix (TOYOBO, Osaka, Japan). The vector was linearized using HindIII and BamHI (NEB, Nebraska, USA). Subsequently, the CDSs of the 4 *ZmACR* genes were subcloned and inserted into the destination vector under the control of the CaMV 35S promoter using the ClonExpress^®^ II One Step Cloning Kit (Vazyme, Nanjing, China). Finally, the vectors containing the target genes and GFP were subsequently transformed into Agrobacterium GV3101, which was subsequently used to infect tobacco leaves. The fluorescence was observed using a Leica TCS SP8 (Mannheim, Germany) confocal microscope imaging system.

### RNA extraction and quantitative real-time PCR of the *ZmACR* genes under salt stress

Total RNA was extracted from leaves using a CWBIO Ultrapure RNA Kit (DNase I) (CWBIO, Beijing, China), and the integrity of the RNA was evaluated via 1.2% agarose gel electrophoresis. First-strand cDNA synthesis was performed using HiScript^®^ III RT SuperMix (+gDNA WIper) (Vazyme, Nanjing, China). Quantitative real-time polymerase chain reaction (qRT-PCR) was conducted in triplicate for each sample using ChamQ SYBR qPCR Master Mix (Low ROX Premixed) (Vazyme, Nanjing, China). The gene-specific primers for qRT-PCR listed in **Supplementary Table S1** were used to amplify *ZmACR* genes by PCR under the following conditions: predenaturation at 95°C for 30 s, denaturation at 95°C for 10 s, annealing at 60°C for 30 s, and 40 cycles (Ge et al., 2022). An ABI 7500 FAST Real-Time PCR System was used for qRT-PCR. Maize ACTIN was used as a control for normalization between samples (**Supplementary Table S1**). Comparative threshold cycling was used to calculate relative transcription levels (Livak and Schmittgen, 2001).

### Transformation of *Arabidopsis* and stress treatment of overexpression plants

After exposure to salt treatments, *ZmACR5* expression in leaves increased the most, approximately 15-fold. These findings suggested that *ZmACR5* may play a crucial role in the response of maize to salt stress. To further investigate the function of *ZmACR5*, we ectopically overexpressed *ZmACR5* in *Arabidopsis* using the PWM101 vector and Agrobacterium-mediated floral-dip method (Clough and Bent, 1998). Two stable overexpression lines (*OE1* and *OE2*) were identified after hygromycin screening and expression identification. The growth conditions and stress treatments were applied to both the transgenic and wild-type plants as previously described (Dong et al., 2012), with minor modifications. Specifically, surface-sterilized seeds harvested from both T3 transgenic lines and the wild type were grown on 1/2 MS agar media for approximately ten days. The uniform plants were transferred to small pots, filled with nutrient soil and vermiculite, in a greenhouse, each containing ten plants. The temperature and light cycle were set at 22°C with a 16 h/8 h (light and dark) photoperiod. After another 7 days of growth, we divided the wild-type and overexpressing plants into three groups. The plants in the control group were watered with regular water, while those in the two treatment groups were watered with 100 or 150 mmol/L salt solution, with 30 ml of solution added to each pot each day. This process continued for 10 consecutive days. Finally, the leaf length and width, as well as the fresh and dry weights above ground, of each plant were measured to evaluate salt tolerance.

### Plant materials and treatment

To grow the maize plants, plump seeds were picked and treated with 75% ethanol for 1 min, followed by sterile water for 6 h. The seeds were subsequently placed on moist sterile filter paper and incubated in the dark at 28°C for approximately 2 days. Once the radicles reached a length of 1-2 cm, the plants were transplanted into small pots filled with nutrient soil and vermiculite (at a ratio of 3:1) for growth in a greenhouse. The temperature and light cycle were set at 26°C with a 14 h/10 h (light and dark) photoperiod. When the maize plants had grown to the two-leaf stage, they were transferred to a hydroponic device and grown in improved Hoagland nutrient solution until the plants reached the four-leaf stage (the nutrient solution was changed every two days). Maize plants with consistent growth were selected and divided into two groups: one group served as a blank control without any treatment, while the other was treated with 250 mmol/L NaCl solution. The maize leaves from both groups were collected at 24 h after salt treatment, immediately frozen in liquid nitrogen and stored in an ultralow temperature refrigerator at -80°C for subsequent experiments.

## Results

### Genome-wide identification and chromosomal localization of ACR genes in maize

We used Pfam (PF01842) to determine the putative members of the ACR gene family in maize. A total of 28 candidate *ZmACR* genes were identified from the B73 genome database. Subsequently, we analyzed various characteristics of these genes, including amino acid length, molecular weight and isoelectric point. The amino acid length of the identified ZmACRs ranged from 105 (ZmACR11) to 620 (ZmACR15). The molecular weights of the products varied from 11513.8 Da (ZmACR11) to 67475 Da (ZmACR15). Additionally, most of the predicted isoelectric points of these 28 ZmACR genes were acidic proteins, except for ZmACR15, ZmACR17 and ZmACR21, whose values ranged from 3.92 to 9.22; these values provided important information for subsequent protein separation, purification and electrophoresis. The detailed information is listed in **Supplementary Table S2**.

Twenty-eight *ZmACR* genes were unevenly distributed on nine chromosomes, with the exception of chromosome 9 (**Supplementary Figure S1**). We assigned numbers to the 28 *ZmACR* genes based on their physical positions, from *ZmACR1* to *ZmACR28*. Most of the *ZmACR* genes were located at the proximal or distal end of chromosomes. Chromosome 4 contained the highest number of *ZmACR* genes (6, approximately 21.4%), followed by chromosomes 1 and 2 (5 each, approximately 17.9%). Chromosome 5 housed 3 *ZmACR* genes, while Chromosome 8 possessed the fewest *ZmACR* genes (1, approximately 3.6%). The remaining chromosomes (3, 6, 7, and 10) contained 2 *ZmACR* genes (approximately 7.1%).

### Phylogenetic tree construction of ACR family genes in maize

**Figure 1:**
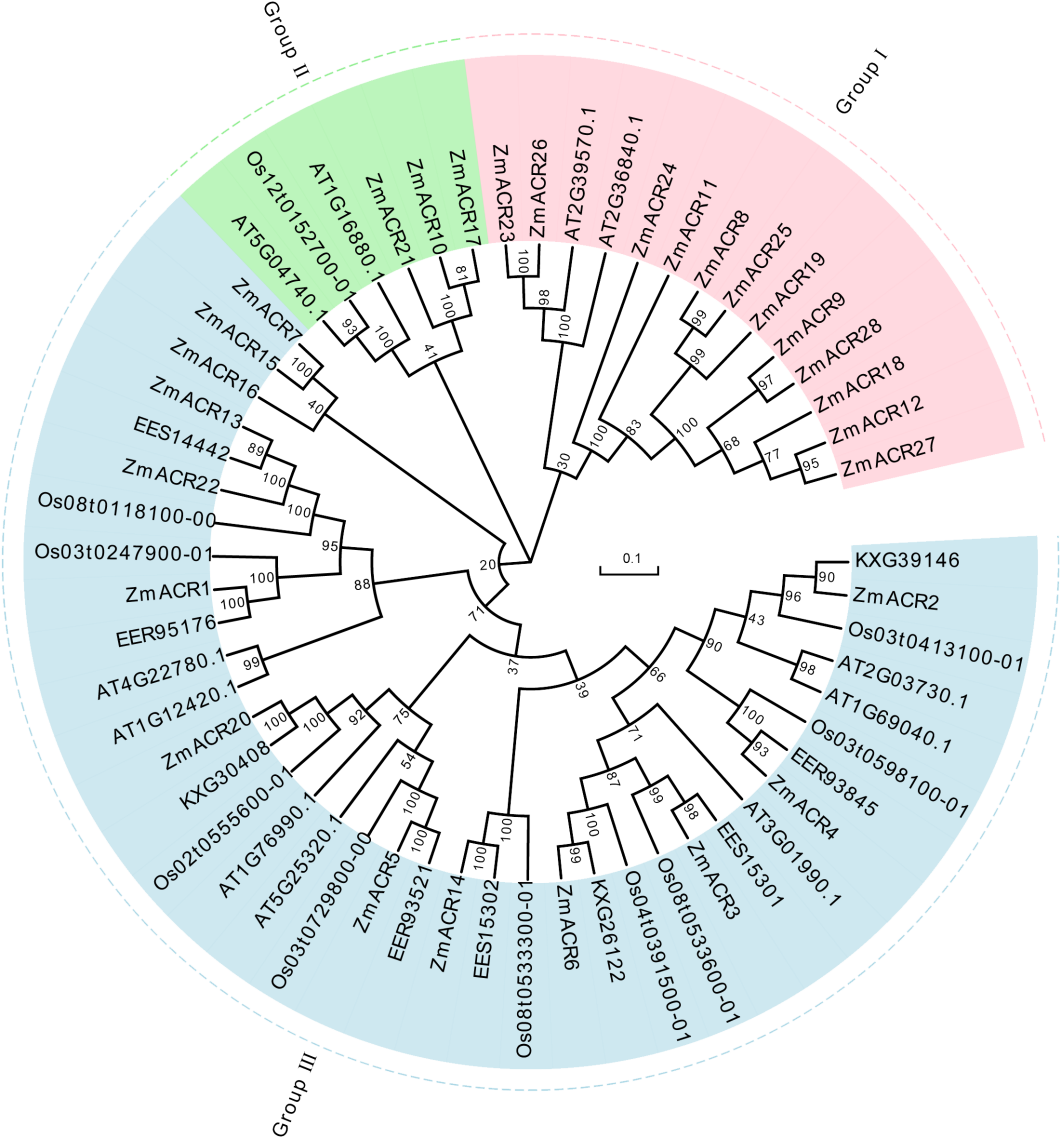
The phylogenetic tree of ACR proteins from *Arabidopsis thaliana*, *Oryza sativa*, sorghum and maize. The picture represents the phylogenetic tree as constructed using amino acid (AA) sequences based on the neighbor-joining (NJ) method where the number on the node represented the bootstrap values. These proteins were clustered into three groups represented by different colors, with each subgroup marked outside the circle as I, II and III. Zm: maize; Os: *Oryza sativa*; the others: sorghum.

To gain further insight into the evolutionary relationship and categorization of ACR genes across different species, 58 ACR genes, including 28 in maize, 11 in *Arabidopsis*, 10 in rice and 9 in sorghum, were used to construct an unrooted phylogenetic tree via the neighbor-joining (NJ) method in MEGA7 software (**Figure 1**). These ACR sequences were subsequently clustered into three subgroups. The largest subgroup, Group III, comprised 13 ACR genes of maize, 9 ACR genes of rice, all 9 ACR genes of sorghum and 7 ACR genes of *Arabidopsis*. Group I consisted of 12 ACR family members of maize and 2 ACR family members of *Arabidopsis*. Group II included 3 ACR genes from maize, 2 ACR genes from *Arabidopsis* and 1 ACR gene from rice. In the evolutionary tree, ACR genes consistently exhibited closer evolutionary relationships between sorghum and maize, both of which were C4 plants. In addition, compared to those in other species, the ACR genes in *Arabidopsis* tended to have separate branches, suggesting that there was a relatively distant evolutionary relationship between *Arabidopsis* and the other three species. This finding suggested that there was divergence of ACR genes between monocotyledonous and dicotyledonous plants during evolution. Furthermore, the number of ACR members in maize was significantly greater than that in the other three species, likely due to the large genome of maize, which led to the expansion of ACR genes during genome evolution.

### Investigation of *ZmACR* gene structures and conserved motifs

To understand the structural features of the *ZmACR* genes, gene structure and conserved motif analyses were also conducted. The results suggested that genes within the same group had similar organization profiles, and most genes contained multiple introns, indicating a particular phylogenetic state. The number and length of untranslated regions (UTRs), exons and introns exhibited significant divergence (**Supplementary Figure S2A**). The genes in Groups I and II contained a greater number of exons, ranging from 5 to 16, while the number of exons in Group III ranged from 3 to 9, except for *ZmACR7* (18) and *ZmACR15* (13), which displayed relatively simple genetic structures. Most of the *ZmACR* genes contained UTRs, although some genes in Groups I (*ZmACR9* and *ZmACR11*) and III (*ZmACR15*) lacked UTRs, which may affect the regulation of gene expression.

In general, similar amino acid sequences could lead to similar gene functions within the same group. To better understand the conserved amino acid sequences of ZmACR proteins, we conducted protein sequence alignment and motif analyses. A total of 10 motifs were identified and labeled after motifs 1 to 10 (**Supplementary Figures S2B, C**). With the exception of ZmACR7 and ZmACR15 in Group III, most of the ZmACR proteins contained conserved motifs, with almost all the genes containing motif 2 and motif 5. The genes in Group II had the fewest motifs, with only Motifs 2 and 9, which was quite different from the other subgroups. All genes in Group I had Motif 2 and Motif 5, which was similar to most genes in Group III. Genes in Group III possessed varying amounts of Motif 2, 5 and 10 combinations. Interestingly, although genes in different subgroups contained different motifs, genes within the same subgroup appeared to share similar motifs. In summary, the conserved motif compositions and distributions of ZmACR proteins exhibited significant differences among the different subgroups, which suggested that these ZmACR proteins might have undergone functional group divergence during evolution.

### Gene duplication, Ka/Ks analysis and collinearity analysis of ZmACR genes in maize

**Figure 2:**
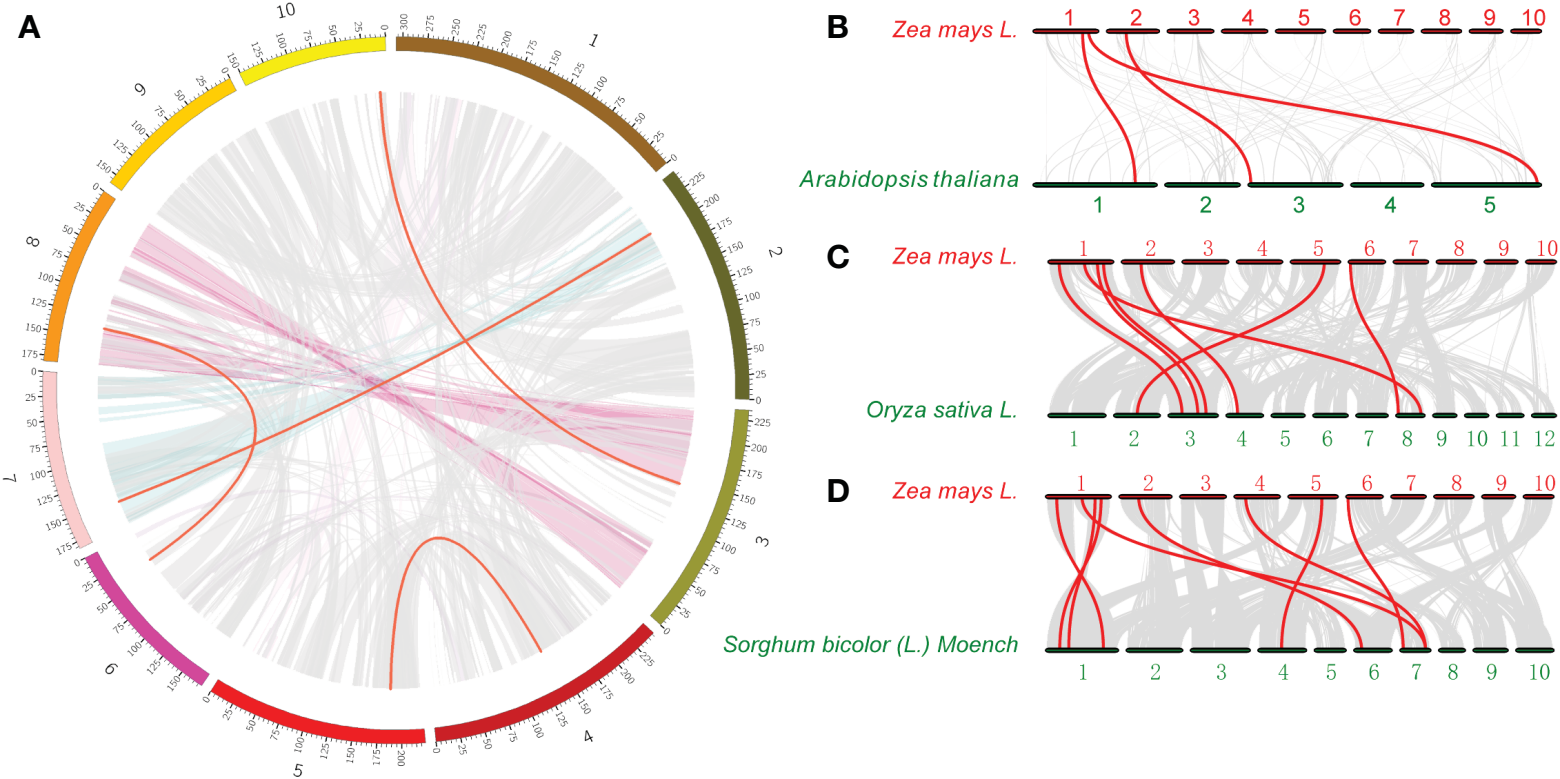
Interchromosomal relationships of *ZmACR* genes and collinearity analysis in maize, *Arabidopsis*, rice, and sorghum. **(A)** Interchromosomal relationships of *ZmACR* genes. Grey lines indicate all syntenic blocks in the maize B73 genome. Red lines indicate collinear blocks of *ZmACR* genes in the maize genome. **(B)** Collinearity of ACR genes between maize and *Arabidopsis.* **(C)** Collinearity of ACR genes between maize and rice. **(D)** Collinearity of ACR genes between maize and sorghum. Red lines indicate orthologous ACR genes.

Genome duplication has long been considered as one of the main drivers of evolution. During gene evolution, tandem repetition and large fragment duplication are the driving forces behind the expansion of gene families within genomes. Previous studies have shown that gene pairs with a sequence similarity of more than 70% and within a distance of 100 kb are considered tandem repeats (Wei et al., 2022a). Interestingly, no tandem duplication events were detected in the ACR gene family in maize. In contrast, 4 pairs of *ZmACR* genes, *ZmACR8*/*ZmACR25*, *ZmACR12*/*ZmACR27*, *ZmACR17*/*ZmACR21* and *ZmACR23*/*ZmACR26*, were identified as segmented repeats (**Figure 2A**). These results suggested that the primary driver of *ZmACR* gene diversity was segmental duplication events.

The ratio of Ka (nonsynonymous substitution) to Ks (synonymous substitution) is a critical parameter that reflects the selection pressure of species (Zhang et al., 2006). To clarify the selection pressure on *ZmACR* genes, we calculated Ka, Ks and their Ka/Ks ratio using KaKs_Calculator2.0 software. The results showed that only two pairs of genes were significantly selected (**Supplementary Table S3**). The Ka/KS ratios of the two gene pairs were significantly less than 1, indicating negative selection and suggesting that the ACR gene family in maize was possibly subjected primarily to purifying selection during the evolutionary process.

To further investigate the evolutionary history of the ACR genes between maize and 3 other species, one dicotyledon (*Arabidopsis*) and two monocotyledons (rice and sorghum), collinearity analysis was performed (**Figures 2B-D**). The collinearity relationships revealed 3, 7 and 8 pairs of ACR genes between maize and *Arabidopsis*, rice, and sorghum, respectively, and 8 *ZmACR* genes were involved, which further confirmed the close evolutionary relationship between maize and sorghum. The number of collinear gene pairs between maize and rice, sorghum was greater than that between maize and *Arabidopsis*, which was similar to the evolutionary relationships between dicots and monocots. These results clearly suggested that ACR genes may have existed before the divergence of monocotyledons and dicotyledons and further verified the evolutionary differences between monocotyledons and dicotyledons.

### Secondary structure and tertiary structure analysis of the ZmACR proteins

The role of proteins is closely linked to their structure. In order to gain a more profound insight into ZmACR proteins, we carefully selected 8 ZmACRs from distinct subgroups for the prediction of their secondary structure and the construction of 3D models (**Supplementary Figure S3**, **Supplementary Table S4**). Our analysis revealed that alpha helices, extended strands, beta turns, and random coils constitute the secondary structure of ZmACR proteins (**Supplementary Table S4**). However, the distribution of these secondary structures varied among different protein subgroups. In Group I (e.g., ZmACR26 and ZmACR27), random coils represented the largest proportion (approximately 40%), followed by alpha helices (32.81%-39.10%) and extended strands (14.05% - 20.73%). Conversely, proteins in Group II, such as ZmACR17 and ZmACR21, predominantly exhibited alpha helices and random coils, accounting for 32.08% ~ 34.99% and 33.1% ~ 38.05%, respectively. The proportion of extended strands in Group II proteins was notably higher compared to Group I and III. In contrast, Group III (e.g., ZmACR3, ZmACR5, ZmACR15, and ZmACR22) showed alpha helices as the major component (36.11% ~ 40.9%), followed by random coils. Beta turns were the least abundant among all secondary structures of ZmACR proteins (**Supplementary Table S4**). Variations in the secondary structure compositions within these ZmACR protein subgroups contributed to the alterations in their three-dimensional structures (**Supplementary Figure S3**). The homology modeling of ZmACR proteins provided a clear view of the composition and arrangement of their secondary structures (**Supplementary Figure S3A**). The GMQE (global model quality estimate) values ranged from 0.66 (ZmACR21) to 0.83 (ZmACR26), with sequence identity between an ACR protein and its homologous model varying from 85.52% to 100%, affirming the accuracy of 3D structure prediction for these ZmACR proteins. The secondary structures of ZmACR proteins, including α-helices, random coils, β-turns, and extended chains, are further intricately folded through the interaction of side chain groups, leading to the formation of a compact spherical spatial structure maintained by various secondary bonds. The stability of the tertiary structure is essential for the biological activity of proteins.

### Subcellular localization prediction of ACR proteins in maize

The protein expression site is closely associated with its function. Therefore, we used the online software CELLO to predict the subcellular localization of ZmACR proteins to gain insight into the potential functions of *ZmACR* genes (**Supplementary Figure S4**). The results showed that the genes in different subgroups exhibited varying expression locations. Specifically, the *ZmACR* genes in Group I were predominantly expressed in the cytoplasm, followed by the nucleus and chloroplast. The three *ZmACR* genes in Group II exhibited a high likelihood of being expressed in chloroplasts and mitochondria. The genes in Group III were predicted to be chiefly expressed in the nucleus and cytoplasm, with chloroplasts and mitochondria being secondary sites. Overall, genes within the same subgroup tended to be expressed at the same sites, suggesting functional similarity.

**Figure 3:**
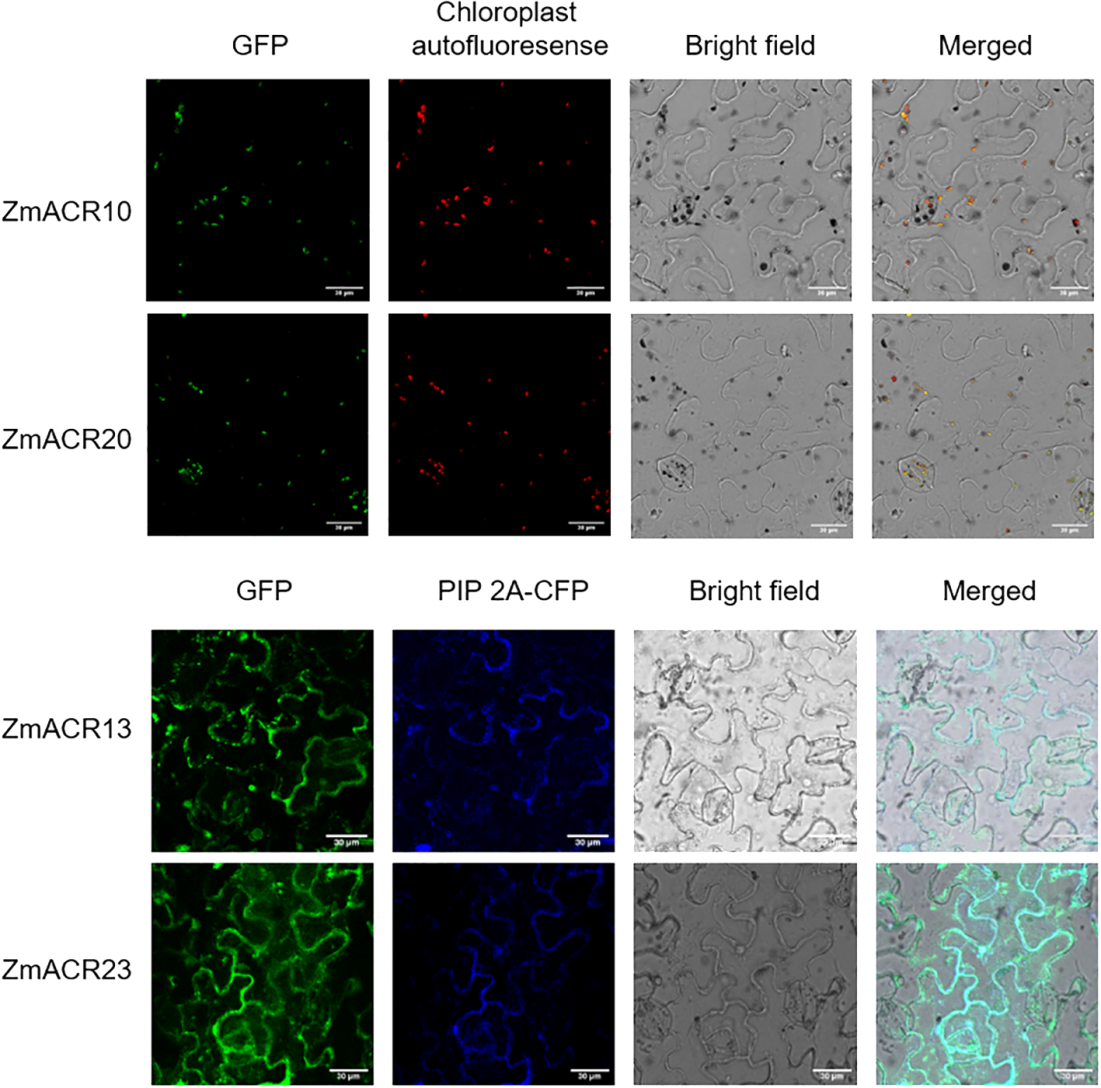
Subcellular localization of ZmACRs in tobacco leaf epidermal cells determined via confocal laser-scanning microscopy. The GFP fluorescence of 4 selected ZmACR fusion proteins, marker fluorescence, bright field and merged images were shown from left to right.

To validate the prediction results, 4 randomly selected *ZmACR* genes from each of the three subgroups were individually attached to a GFP tag and driven by the CaMV 35S promoter. These constructs were then transiently expressed in the epidermal cells of *Nicotiana benthamiana* leaves to determine their subcellular localization. In *N. benthamiana* leaf epidermal cells, fluorescence signals corresponding to the GFP control were observed in almost all parts of the cells, suggesting normal expression of the GFP (**Supplementary Figure S5**). The GFP fluorescent signals of the ZmACR10 and ZmACR20 fusion proteins were exclusively detected in the chloroplast and were not observed in the cytoplasm, indicating that these two proteins may be localized in the chloroplast (**Figure 3**). The GFP signals of the ZmACR13 and ZmACR23 fusion proteins not only colocalized with the cyan membrane-anchored marker PIP 2A-CFP after transient expression in tobacco leaves but also extended to the inner part of the cell membrane, suggesting that both genes were likely localized to the cytoplasm (**Figure 3**). In addition, the ZmACR13 fusion protein was also found to be localized to the nucleus, as evidenced by the presence of several dot distributions of the GFP fluorescence signal. These experimental results were largely consistent with the predicted results, thereby confirming the reliability of the predictions.

### Analysis of *cis*-acting elements of *ZmACR* promoters

*Cis*-acting elements play pivotal roles in regulating transcription during numerous physiological processes in plants, such as plant growth, development, and biotic and abiotic stress responses. To explore the potential molecular function of the *ZmACR* gene family, the 1.5 kb promoter sequence of each *ZmACR* gene was extracted and submitted to PlantCARE to predict the *cis*-acting elements. In total, 81 distinct *cis*-acting regulatory elements associated with plant growth, development and abiotic stress responses were identified (**Supplementary Figure S6**, **Supplementary Table S5**). Several fundamental elements, such as the CAAT-box and TATA-box, were present in the majority of *ZmACR* promoters. Moreover, many *ZmACR* promoters contained *cis*-acting elements associated with light reactions (e.g., AE-box, G-box, GA-Motif, GATA motif, GT1-motif, and TCT-motif), hormone response elements (e.g., ABRE, STRE and MYB) and drought induction elements (e.g., MBS and W-boxes). These findings suggested that *ZmACR* genes might participate in hormone signaling pathways, biotic or abiotic stress responses, and other physiological processes.

### Expression profiles of *ZmACR* genes in different tissues

**Figure 4:**
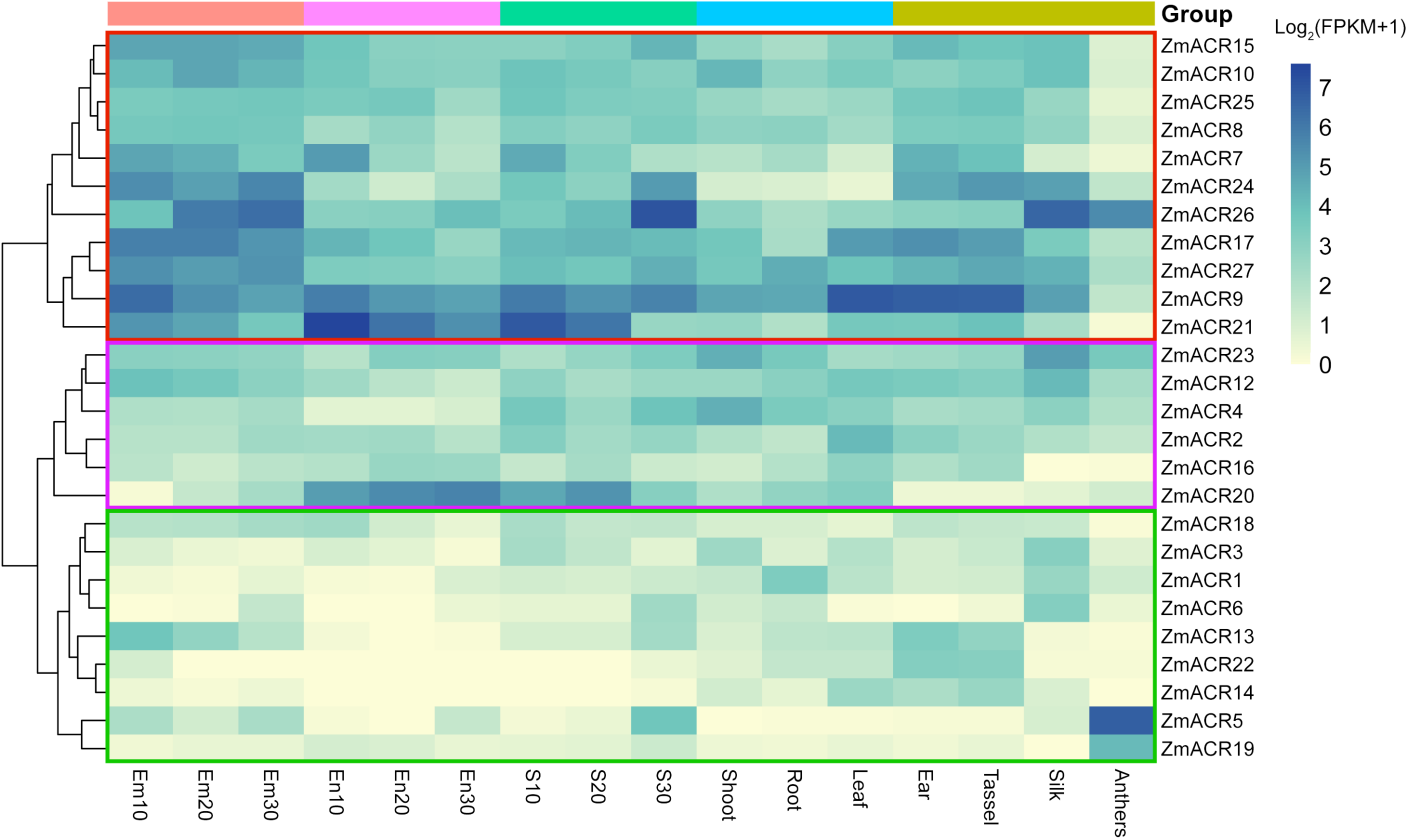
Expression profiles of *ZmACR* genes in maize different tissues. Em indicates the embryo; Em10, Em20 and Em30 indicate that embryo samples were taken 10, 20 and 30 days after pollination, respectively. En represents endosperm; En10, En20 and En30 represent that the endosperm samples were taken 10, 20 and 30 days after pollination, respectively. Seed samples S10, S20 and S30 were taken 10, 20 and 30 days after pollination, respectively.

To further investigate the potential functions of the *ZmACR* genes in maize growth and development, we analyzed the expression levels of 26 *ZmACR* genes (excluding *ZmACR11* and *ZmACR28*) in 10 different tissues, namely, the embryo, endosperm, seed, root, shoot, leaf, ear, tassel, silk and anther tissues, using the transcriptome data of maize B73 lines from a previous study (Chen et al., 2014). Our findings revealed three distinctive expression patterns among the *ZmACR* genes (**Figure 4**). First, the genes in the red box, such as *ZmACR9*, *ZmACR26*, and *ZmACR17*, were highly expressed in multiple tissues. Second, the genes in the purple box, such as *ZmACR12* and *ZmACR4*, were moderately expressed in different tissues. Third, the genes in the green box were expressed in only a few specific tissues at moderate or low levels. For instance, *ZmACR1* was moderately expressed in the roots but exhibited very low expression in other tissues (**Figure 4**). The first two categories exhibited constitutive expression characteristics. Considering that many ACR genes in plants have been shown to be involved in regulating amino acid metabolism, which is crucial for the growth and development of different plant tissues, *ZmACR* genes might play a role in regulating plant growth and development in various tissues. Notably, the last category of *ZmACRs* exhibited specific tissue expression patterns, indicating that these genes might have unique functions in specific tissues.

### Expression patterns of *ZmACR* genes under salt stress

**Figure 5:**
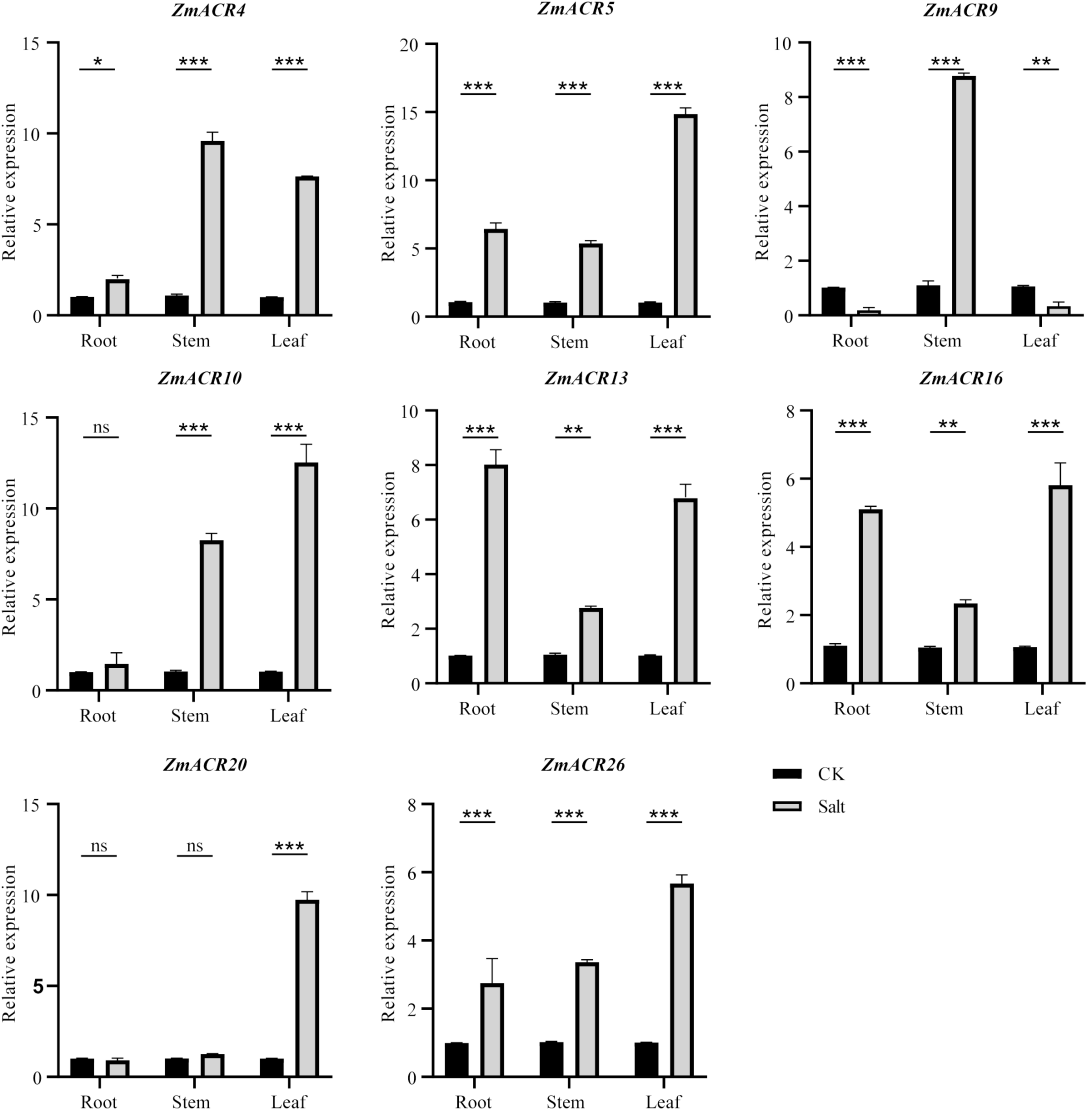
qRT-PCR analysis of *ZmACRs* under salt treatment (250 mmol/L NaCl solution). Data represent mean ± standard error (SE) of three biological replicates. *, **, *** and ****, represent significant differences at P < 0.05, P < 0.01, P < 0.001 and P < 0.0001, respectively. ‘ns’ means not significant. Student’s *t-test* was used to analyze the differences in the relative expression of ZmACR genes between the salt treatment and control groups.

Previous studies have demonstrated that *ZmACR* genes are involved in the response to abiotic stresses and plant hormones in *Arabidopsis* (Singh et al., 2018). The identification of *cis*-acting elements associated with the abiotic stress response at *ZmACR* promoters also indicated the potential role of these elements in the response to abiotic stresses. To further understand the function of *ZmACR* genes under abiotic stress conditions, we randomly selected 8 *ZmACR* genes from different subgroups and performed qRT-PCR to quantify their expression levels under salt stress (**Figure 5**). Compared with those in the control group, 5 out of the 8 ACR genes (*ZmACR4*, *ZmACR5*, *ZmACR13*, *ZmACR16* and *ZmACR26*) exhibited increased expression in roots, stems and leaves after 12 hours of salt treatment, suggesting their potential involvement in the response to salt stress. In particular, the expression level of *ZmACR5* in leaves was upregulated the most, approximately 15-fold, indicating that *ZmACR5* plays a significant role in the response to salt stress. Conversely, the expression level of *ZmACR9* in roots and leaves significantly decreased under salt conditions. Interestingly, the expression of *ZmACR9* in the stems significantly increased eightfold, suggesting that it may play a potential role in maize stem development. In addition, while the expression levels of *ZmACR10* in roots and *ZmACR20* in roots and stems did not significantly differ after salt treatment, their expression levels in stems or leaves significantly increased. In conclusion, these findings revealed that most *ZmACR* genes were involved in the response to salt stress but might operate through distinct mechanisms due to their varied expression patterns.

### The overexpression of *ZmACR5* gene in *Arabidopsis* declined the salt tolerance

**Figure 6:**
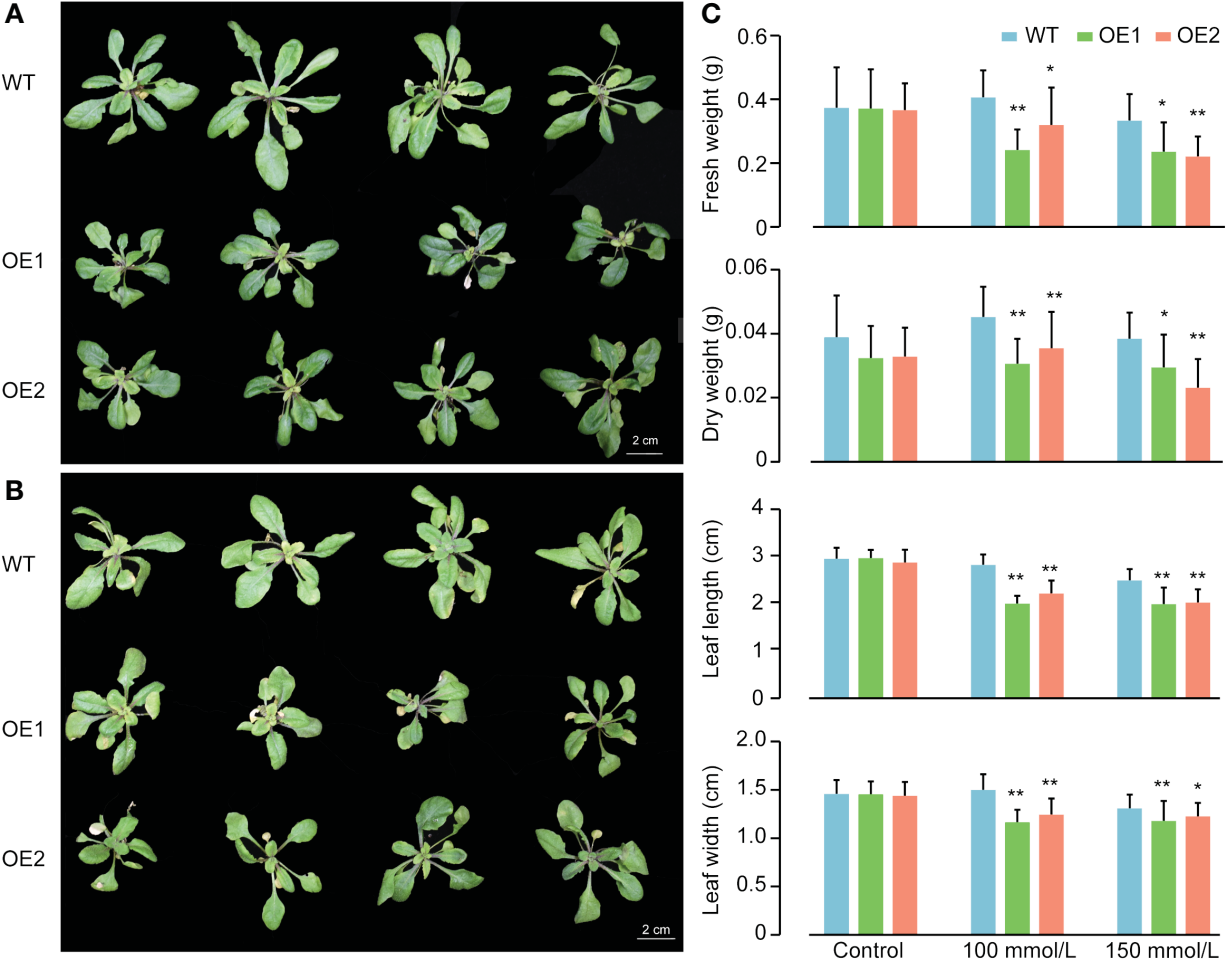
Phenotypes of wild type, *OE1* and *OE2* plants of *ZmACR5* in response to salt stress. **(A)** Salt tolerance in wild type, *OE1* and *OE2* plants at 100 mmol/L NaCl concentration. **(B)** Salt tolerance in wild type, *OE1* and *OE2* plants at 150 mmol/L NaCl concentration. **(C)** Measurement of fresh and dry weight of the above-ground plants, leaf length and width of wild type, *OE1* and *OE2* plants under control, 100mmol/L and 150 mmol/L NaCl concentrations. Bar, mean±SE, cm.

In light of the notable increase in *ZmACR5* expression under salt stress conditions, we ectopically overexpressed this gene in *Arabidopsis* to further investigate its role in the response to salt stress. Through hygromycin screening and qRT-PCR, we successfully identified two stable T3 generation overexpression plants (*OE1* and *OE2*), whose expression levels were approximately 620- to 259-fold greater than those of the wild type (**Supplementary Figure S7**). We subsequently assessed the salt tolerance of the wild-type, *OE1* and *OE2* plants at two NaCl concentrations, 100 and 150 mmol/L. Under normal conditions, there were no significant differences in growth between wild-type *Arabidopsis thaliana* and the two overexpression lines (**Figure 6C**; **Supplementary Figure S8**). However, when exposed to salt stress, both the *OE1* and *OE2* plants exhibited diminished salt tolerance compared to that of the wild type plants at both 100 mmol/L and 150 mmol/L (**Figures 6A, B**). Specifically, compared with those of the control plants, the leaf length and width of both the *OE1* and *OE2* plants were significantly lower under the 100 mmol/L salt treatment, with leaf length decreasing by 0.8 and 0.6 cm and leaf width decreasing by 0.34 and 0.26 cm, respectively. Additionally, the fresh weight and dry weight of the above-ground plants also exhibited significant decreases under the 100 mmol/L salt treatment, with decreases of 0.165 g and 0.087 g for fresh weight, and 0.015 g and 0.010 g for dry weight, respectively (**Figure 6C**). The same phenotypes were observed at a salt concentration of 150 mmol/L, albeit to varying degrees (**Figure 6C**). These observations indicated that plants overexpressing the *ZmACR5* gene were more susceptible to salt stress than the wild type plants. Therefore, we confirmed that *ZmACR5* can negatively regulate salt stress in *Arabidopsis*.

## Discussion

Genes containing the ACT domain (ACR genes) are widely distributed in microorganisms, animals and plants. While many ACR genes have been studied and characterized in various species, little is known about them in maize (Chipman and Shaanan, 2001; Hsieh and Goodman, 2002; Grant, 2006; Hayakawa et al., 2006; Mas-Droux et al., 2006). Advancements in sequencing technology have led to the release of a large amount of genomic data (Gore et al., 2009; Chia et al., 2012; Bukowski et al., 2018), enabling us to analyze the characteristics and functions of gene families. In the present study, we identified 28 *ZmACR* genes in maize that contained complete ACT-specific domains and analyzed their gene structure and molecular characteristics. The proteins encoded by these ACR genes in maize exhibited diverse physicochemical properties and significant variations in size, function, and regulatory mechanisms, but all shared at least one stable ACT domain.

Evolutionary analysis provides insights into the relationships and origins of gene family members during the evolutionary process (Li et al., 2021; Wei et al., 2022b). Based on the phylogenetic tree, 3 branches were found among the 58 ACR proteins in maize, sorghum, rice and *Arabidopsis*, each characterized by different gene numbers and structures. Differences in gene structure are closely correlated with gene evolution (Cao and Shi, 2012). During the evolution of this gene family, a clear divergence occurred between monocotyledonous and dicotyledonous plants and between maize and sorghum, the two C4 plants, revealing relatively close evolutionary relationships among ACR proteins. Furthermore, we discovered that phylogenetic classification was closely associated with gene structure. Most ZmACR proteins in the same subgroup shared common motifs, similar gene structures and specific arrangements and combinations of different motifs.

Gene amplification plays a crucial role in the evolution of genomes by giving rise to new functional genes and facilitating the differentiation of new species, thus increasing plant adaptability to harsh environments during evolution (Chow et al., 2012). Repeated sequences are abundant in the genomes of various species, with identical segments known as repeat units. These repeat units constitute a significant portion of the entire plant genome, ranging from 10% to 85% (Wendel et al., 2016). In maize, for instance, approximately 85% of the genome is composed of transposon element repeats (Schnable et al., 2009). Polyploidization, a process that results in the duplication of chromosome segments, preserves multiple duplicate segments in the genome, in turn leading to fragment duplication. For instance, within the maize ACR gene family, 2 pairs of genes were identified as segmented repeat genes, suggesting that fragment repetition is a key driver of the evolution of ACR genes in maize. Additionally, the evolutionary process of these *ZmACR* genes appeared to have undergone purifying selection, as indicated by Ka/Ks analysis.

The location of proteins within cells is closely associated with their function. Therefore, understanding the subcellular localization of proteins is crucial for functional research. In *Arabidopsis*, among the identified ACR genes, ACR1 was predicted to be a nuclear protein, while the remaining seven ACR proteins were cytoplasmic proteins (Hsieh and Goodman, 2002). The recently discovered *Arabidopsis* ACR proteins ACR11 and ACR12 were expressed in chloroplasts, with ACR11 specifically expressed in green tissues (Sung et al., 2011). In our study, we predicted that ZmACR proteins were primarily expressed in the cytoplasm, nucleus and chloroplast. Experimental evidence has revealed that ZmACR10 and ZmACR20 are expressed in chloroplasts, while ZmACR13 and ZmACR23 are most likely localized in the cytoplasm. Notably, the GFP fluorescence of the latter two fusion proteins exhibited spot-like signals, making it challenging to determine whether these signals were noise or indicative of alternative localization signals. Since the majority of the amino acids are synthesized in the cytoplasm, it is conceivable that some of the ZmACR proteins may function as regulatory proteins involved in amino acid metabolism or signal transduction. In plants, ACR proteins likely play positive or negative regulatory roles and may have regulatory effects on their respective target proteins or enzymes.

The identification of upstream *cis*-acting elements of each gene provided valuable information for the regulatory mechanisms potentially governing the ACR gene family. These *cis*-acting elements typically bind to transcription factors to regulate downstream gene expression (Huang et al., 2018; Tian et al., 2019). Examination of *ZmACR* gene promoters revealed many light-responsive elements, such as G-boxes, hormone-related elements (jasmonic acid and abscisic acid-responsive elements) and abiotic stress elements. Previous studies have demonstrated that G-boxes are responsive to light and regulate plant flowering (Menkens et al., 1995). The jasmonic acid signaling pathway is involved in the regulation of plant salt stress responses (Ma et al., 2006; Geng et al., 2013). The uneven distribution of two jasmonic acid-related elements, CGTCA-motif and TGACG-motif, on the promoter of the *ZmACR* genes suggested their potential involvement in the salt stress response. Furthermore, the *cis*-acting element ABRE can bind to transcription factors to promote or inhibit the expression of ABA-induced genes (Narusaka et al., 2003) and has been implicated in plant stress resistance, including salt stress resistance, in *Arabidopsis* (Narusaka et al., 2003; Pan et al., 2020). Additionally, the expression patterns of the ACR gene family in *Arabidopsis* were analyzed under adverse stress conditions. After salt treatment, the expression levels of eight ACR genes exhibited varying degrees of change, with ABA and NaCl treatment significantly increasing the expression level of *AtACR8* (Hsieh and Goodman, 2002). These findings suggested that the functions of *ZmACR* genes might be diverse and potentially involved in salt stress. Consequently, 8 *ZmACR* genes from different subgroups were randomly selected for further analysis of their expression under salt conditions. The results demonstrated that these genes indeed responded to salt stress by upregulating or downregulating their expression in roots, stems and leaves. Subsequently, we ectopically expressed *ZmACR5* in *Arabidopsis*. The performance of the two stable T3 generation *ZmACR5*-overexpressing plants was significantly worse than that of the wild-type plants under the two salt concentrations (100 and 150 mmol/L). Notably, compared with those of wild-type plants, the leaf length, leaf width, and fresh and dry weight of aboveground plants significantly decreased, indicating that *ZmACR5* might exert a negative regulatory effect on salt stress in *Arabidopsis*. Taken together, these findings substantiate the significant role of the *ZmACR* genes in the response to salt stress.

## Conclusion

In this study, we comprehensively investigated the maize ACR gene family. Through genome-wide identification, we successfully identified a total of 28 *ZmACR* genes. Subsequently, a range of bioinformatics, qRT-PCR and overexpression analyses were employed to investigate various aspects of these genes, including their gene structure, evolutionary relationships, subcellular localization, collinear gene pairs, *cis*-acting elements, and expression patterns under salt stress. Furthermore, we further elucidated the significance of the ACR gene family in the salt stress response by conducting overexpression experiments with *ZmACR5* in *Arabidopsis thaliana*. These findings highlighted the crucial role of ACR genes in mediating the response to salt stress and contributed significantly to our understanding of the molecular function of ACR genes in maize, facilitating further research in this field.

## Data availability statement

The original contributions presented in the study are included in the article/**Supplementary Material**, further inquiries can be directed to the corresponding author/s.

## Author contributions

HF: Methodology, Writing – original draft, Writing – review & editing. TS: Data curation, Formal analysis, Visualization, Writing – original draft. HG: Data curation, Formal analysis, Visualization, Writing – original draft. JC: Validation, Writing – original draft. YQ: Validation, Writing – original draft. YL: Validation, Writing – original draft. MS: Writing – review & editing. JY: Visualization, Writing – review & editing. PL: Project administration, Supervision, Writing – review & editing. BW: Project administration, Supervision, Writing – review & editing.
