# Supplementary material for: Identification and characterization of ACR gene family in maize for salt stress tolerance
Source: Front Plant Sci. 2024 Apr 30;15:1381056. doi: 10.3389/fpls.2024.1381056 (PMC11091409; doi:10.3389/fpls.2024.1381056)
Supplement: Supplementary file 1 [file DataSheet_1.docx]

**Identification and characterization of ACR gene family in maize for salt stress tolerance**

Hui Fang ^1, †^, Tingyu Shan^1, †^, Haijing Gu ^1, †^, Junyu Chen ^1^, Yingxiao Qi ^1^, Yexiong Li^1^, Ying Wu ^1^, Junyi Li ^1^, Hanqiu Ge ^1^, Muhammad Saeed ^2^, Jinchao Yuan ^3^, Ping Li ^1, *^, Baohua Wang ^1, *^

^1^ Ministry of Agricultural Scientific Observing and Experimental Station of Maize in Plain Area of Southern Region, School of Life Sciences, Nantong University, Nantong 226019, Jiangsu, People’s Republic of China

^2^ Department of Agricultural Sciences, Government College University, Faisalabad, Pakistan.

^3^ Qidong Ruichao Farm, Nantong 226200, Jiangsu, People’s Republic of China

^†^ These authors contributed equally to this work.

^*^ Author to whom correspondence should be addressed.

The following Supporting Information is available for this article:

**Figure. S1** Chromosomal location of *ZmACR* genes in maize. The number on the left of the chromosome is the physical position of genes.

**Figure. S2** Gene structure and conserved motif analysis of *ZmACR* genes in maize. (A) The exon-intron distribution of the 28 *ZmACR* genes. Exons were represented by blue rectangles, introns were represented by black lines, and pink rectangles represented UTR regions (B) The conserved motifs of *ZmACRs*. The various rectangles with divergent colors indicate the different conserved motifs.

**Figure. S3** Three-dimensional (3D) models of 8 selected ZmACR proteins

**Figure. S4** Prediction of the subcellular localization of ZmACR proteins. The color and the size of the circles indicated the reliability of the prediction results. The name of each protein was shown on the right. The site name for the predicted subcellular localization of each ZmACR protein was shown at the bottom.

**Figure. S5** The GFP expression in tobacco leaf epidermal cells after treatment with the empty vector determined via confocal laser-scanning microscopy.

**Figure. S6** *Cis*-acting elements in the *ZmACR* gene promoters. The different cis-acting elements were shown in different colors. The name of each gene was shown on the left. Promoter sequence length was displayed in proportion.

**Figure. S7** Expression identification of 2 *Arabidopsis* plants overexpressing the *ZmACR5* gene.

**Figure. S8** Phenotype of wilt type *Arabidopsis*, and 2 *Arabidopsis* plants overexpressing the *ZmACR5* gene under control.

**Table S1** Primer sequences used in this study.

**Table S2** Protein characteristic information of *ZmACR* family genes in maize.

**Table S3** Statistics of the Ka/Ks analysis.

**Table S4** The secondary structure statistics of the selected ZmACR proteins.

**Table S5** Detailed information of cis-acting regulation elements of *ZmACR* promoters


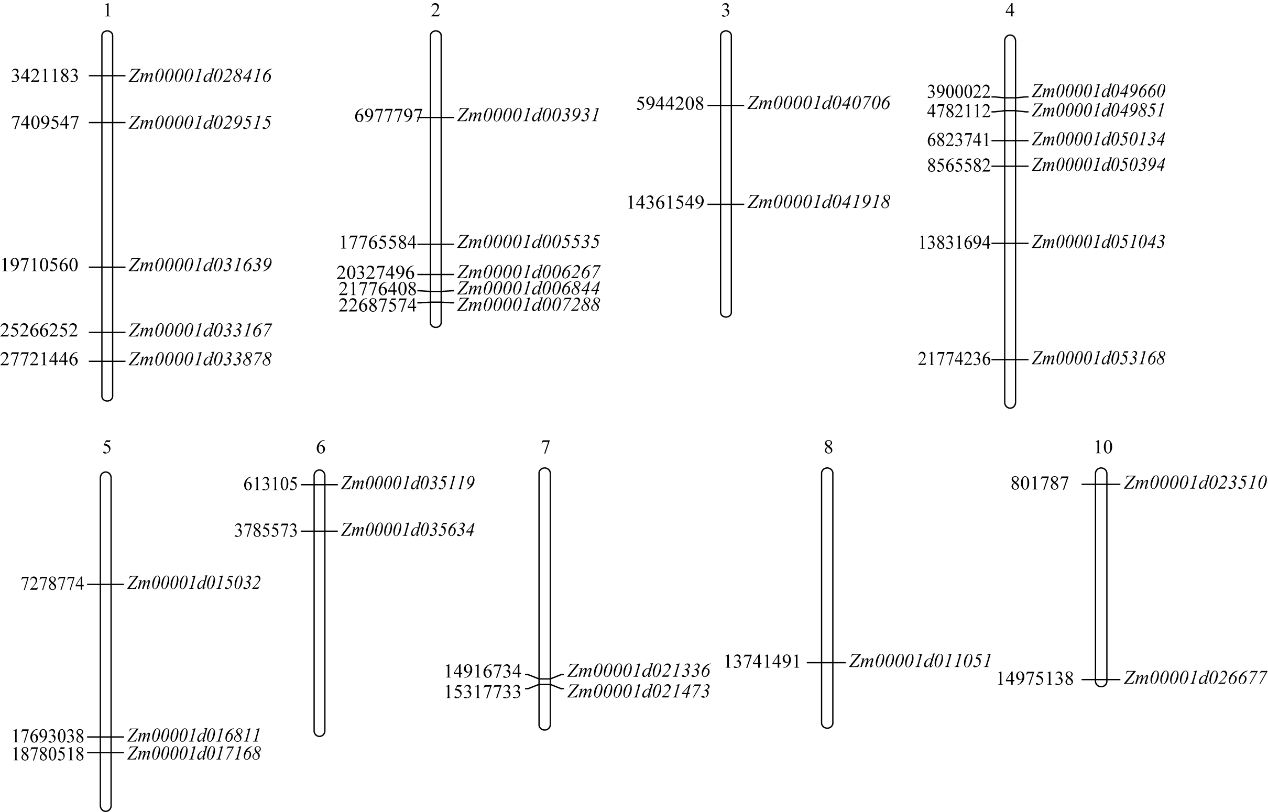


**Figure. S1**. Chromosomal location of *ZmACR* genes in maize. The number on the left of the chromosome is the physical position of genes.


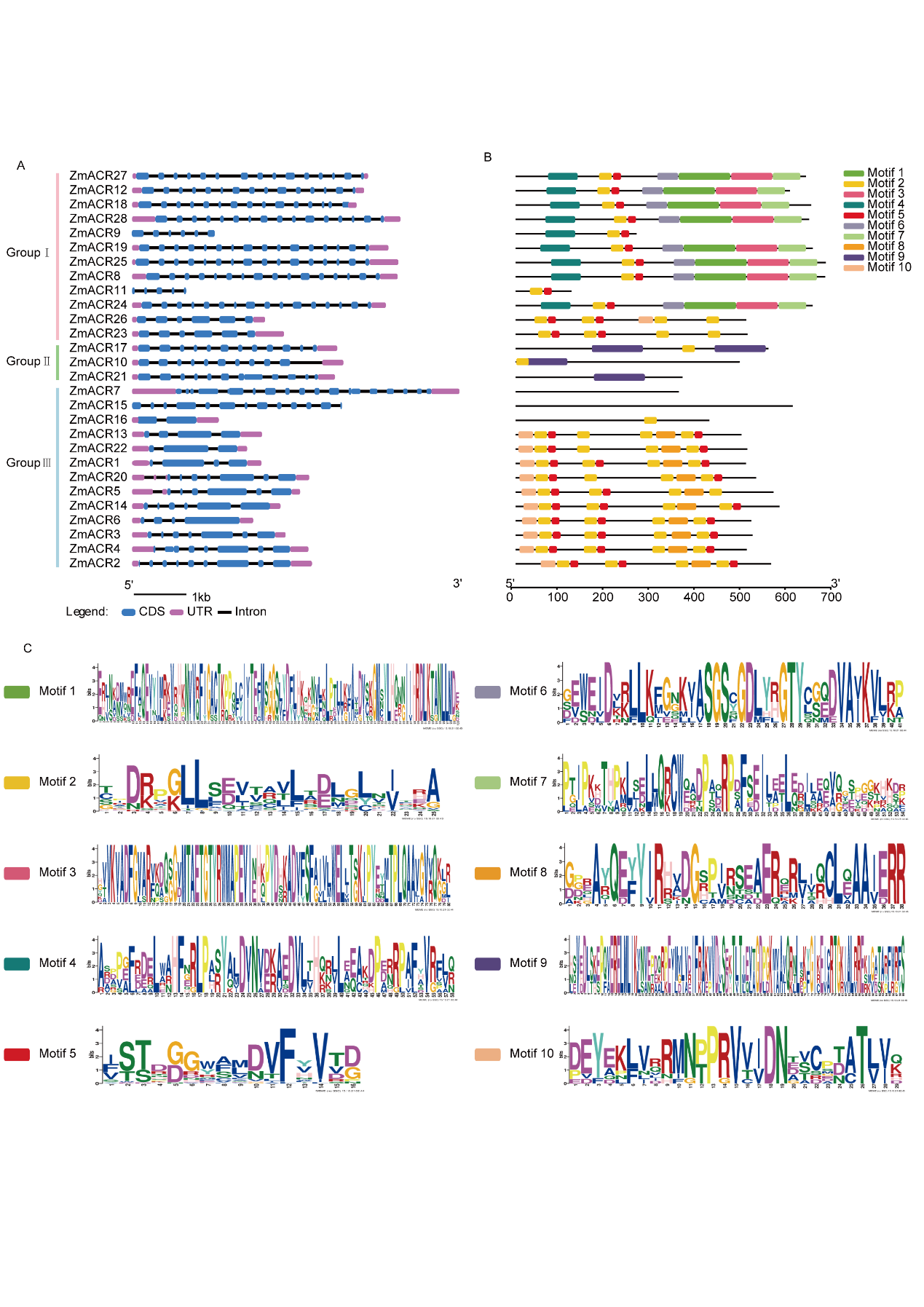


**Figure. S2**. Gene structure and conserved motif analysis of *ZmACR* genes in maize. (A) The exon-intron distribution of the 28 *ZmACR* genes. Exons were represented by blue rectangles, introns were represented by black lines, and pink rectangles represented UTR regions (B) The conserved motifs of *ZmACRs*. The various rectangles with divergent colors indicate the different conserved motifs. (C) The detailed information about 10 motifs of *ZmACRs*.


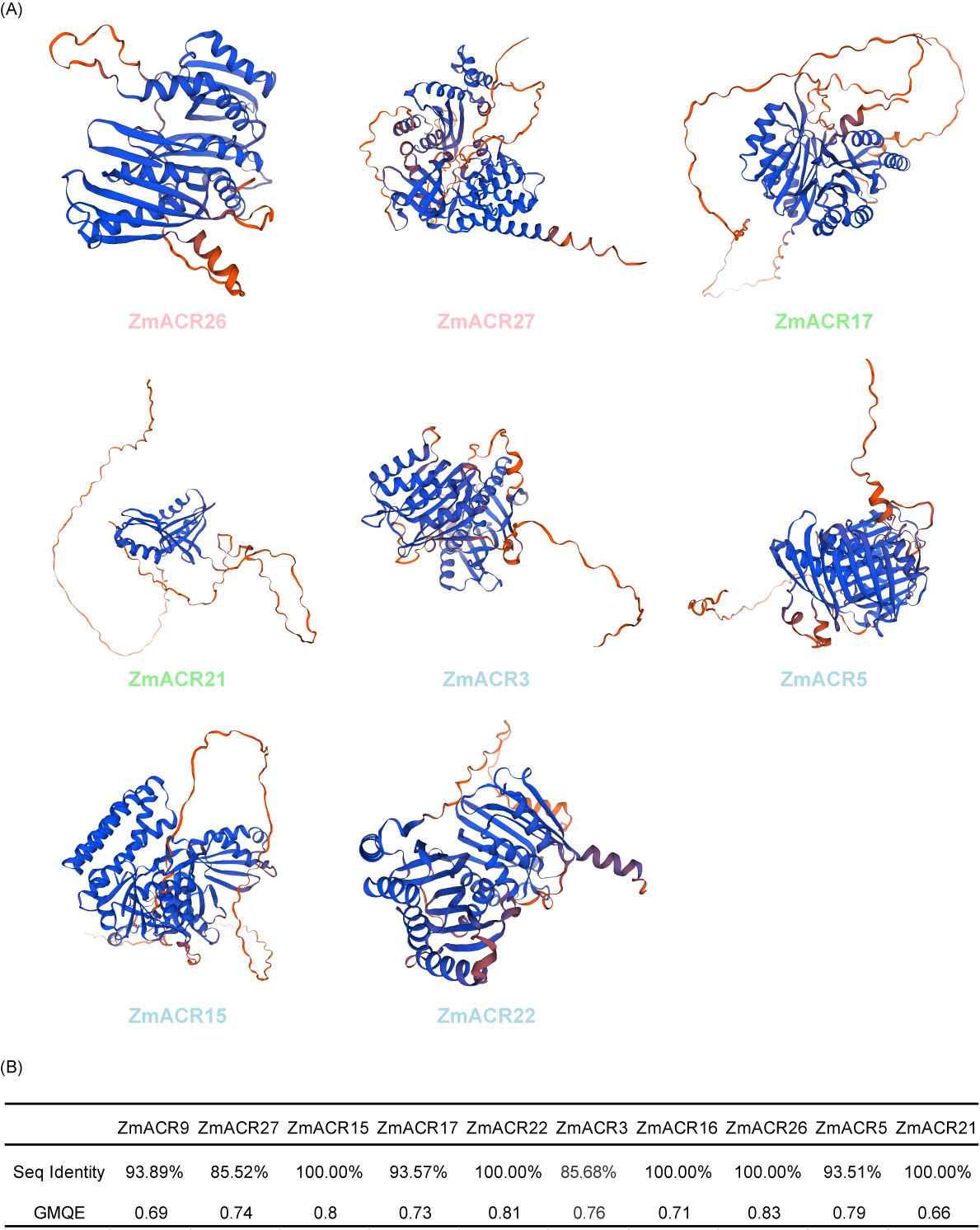


Figure. S3 Three-dimensional (3D) models of 8 selected ZmACR proteins. (A) 3D models prediction of 8 ZmACR proteins. The genes written in lightpink are from Group Ⅰ, written in bright green are from GroupⅡ, and written in skyblue are from Group Ⅲ. (B) The sequence identity and GMQE values of the prediction results. Sequence identity shows the sequence similarity between an ACR protein and its homologous model. GMQE (global model quality estimate) value shows the quality of model, whose value is between 0 and 1, and the closer to 1, the better the modeling quality.


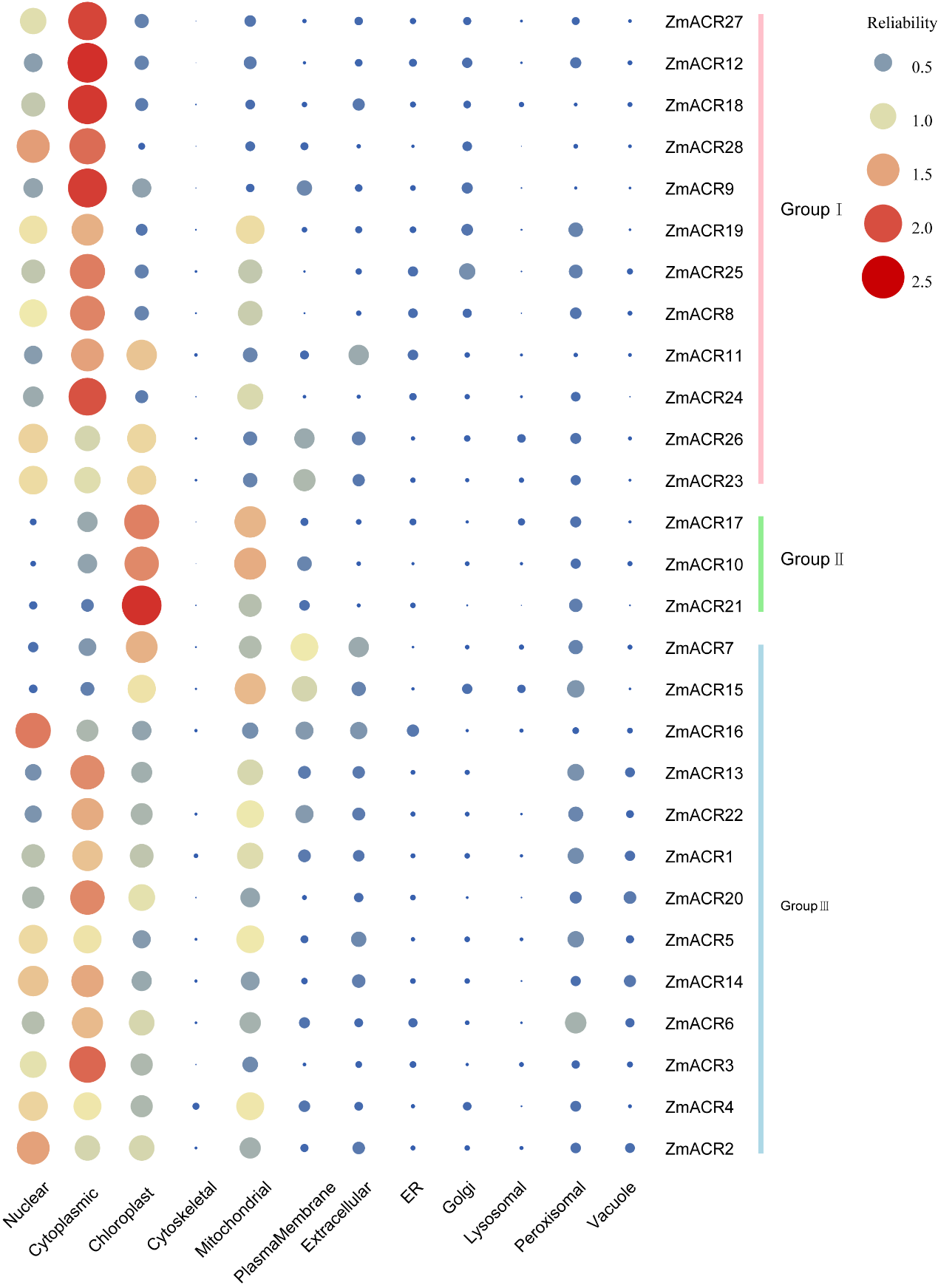


**Figure. S4**. Prediction of the subcellular localization of ZmACR proteins. The color and the size of the circles indicated the reliability of the prediction results. The name of each protein was shown on the right. The site name for the predicted subcellular localization of each ZmACR protein was shown at the bottom.


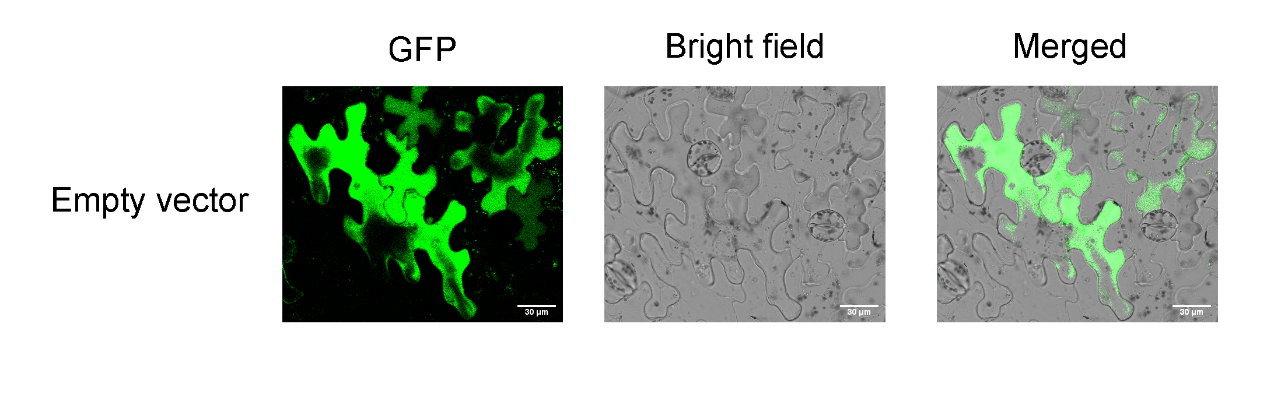


**Figure. S5**. The GFP expression in tobacco leaf epidermal cells after treatment with the empty vector determined via confocal laser-scanning microscopy.


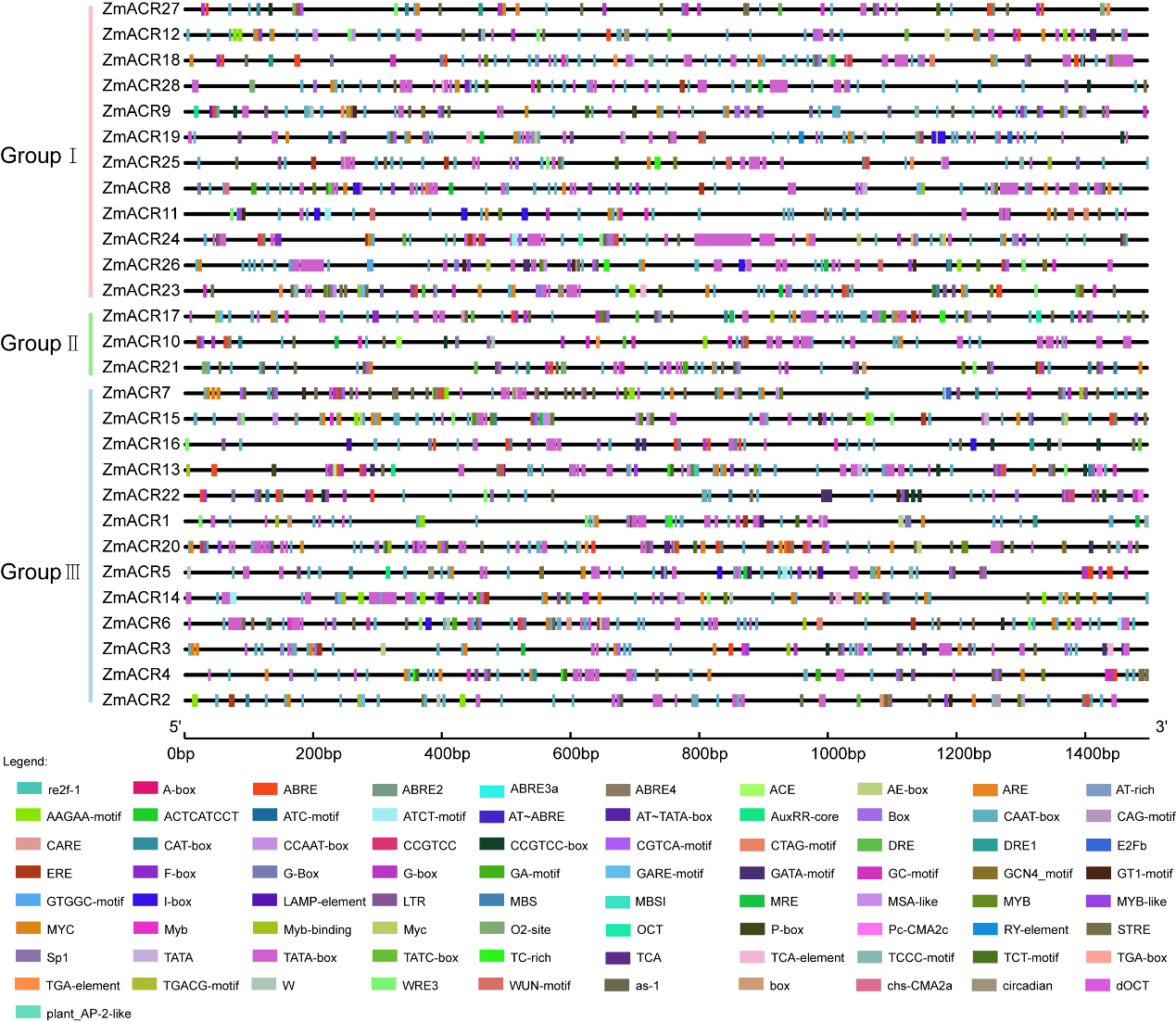


**Figure. S6**. *Cis*-acting elements in the *ZmACR* gene promoters. The different *cis*-acting elements were shown in different colors. The name of each gene was shown on the left. Promoter sequence length was displayed in proportion.


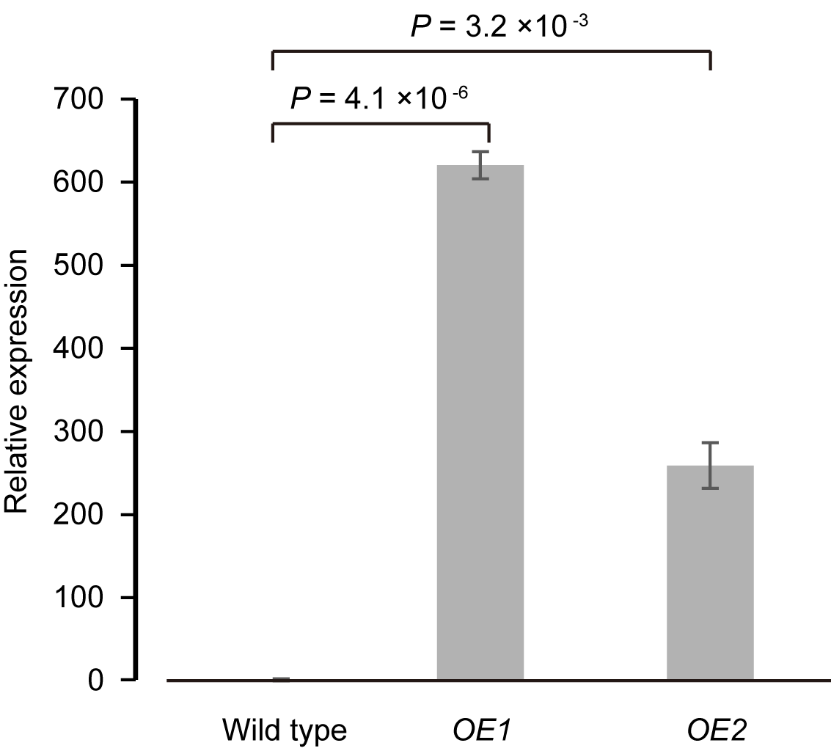


**Figure. S7**. Expression identification of 2 *Arabidopsis* plants overexpressing the *ZmACR5* gene.


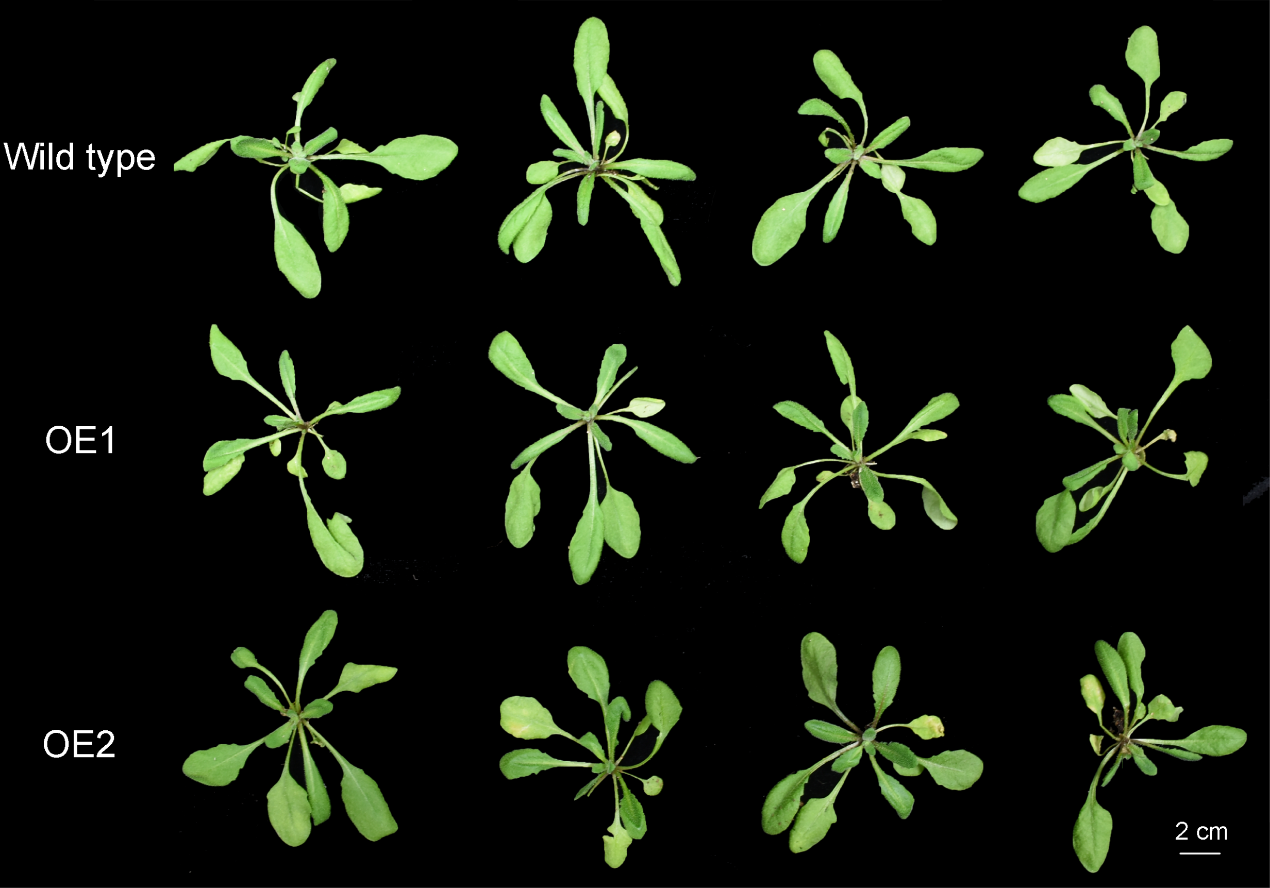


**Figure. S8**. Phenotype of wilt type *Arabidopsis*, and 2 *Arabidopsis* plants overexpressing the *ZmACR5* gene under control.

**Table S1**. Primer sequences used in this study

|  | Primer name | Gene name | Primer sequence（5’-3’ |
| --- | --- | --- | --- |
| qRT-PCR | ZmActin-F | ZmActin8 | GCATCCATGAGACCACCTACAAC |
|  | ZmActin-R |  | GATGGACCCTCCTATCCAGACAC |
|  | Zm33878-F | ZmACR5 | GGACTTCGACCCGCTACT |
|  | Zm33878-R |  | CGGACGTGATGGAGGACA |
|  | Zm33167-F | ZmACR4 | ACGATGGCGACTACGAGC |
|  | Zm33167-R |  | GGATGACTCGCAACCTCT |
|  | Zm49660-F | ZmACR13 | GAGGTGTTCGCCGTGCTG |
|  | Zm49660-R |  | GGTCACCACGGAGTAGCC |
|  | Zm06844-F | ZmACR9 | GGTTGTGGGTGACTATGT |
|  | Zm06844-R |  | AAATCACCTTTGTATCCC |
|  | Zm50394-F | ZmACR16 | ATGGTGTCCGGCCTTCTG |
|  | Zm50394-R |  | GGCGAGGTGGCTGTTGAT |
|  | Zm07288-F | ZmACR10 | ATAATGGGTTTGGTTCGC |
|  | Zm07288-R |  | GCTATCTTGCCAGTTCTA |
|  | Zm11051-F | ZmACR26 | AGGACGATGAAGGACTGC |
|  | Zm11051-R |  | TTCTTCCCGTCTACTTGC |
|  | Zm16811-F | ZmACR20 | TCGTCCGAAGCCAACTAC |
|  | Zm16811-R |  | CTCGGATACTCTTCTGGA |
| Overexpression in *Arabidopsis* | OE-F | ZmACR5 | TCGAGCTTTCGCGAGCTCGGTACCGGTACCATGAAGTACGTCTCCGGGCC |
|  | OE-R |  | GCATGCCTGCAGGTCGACTCTAGATCTAGATCAAGACCTGATGTTGTTGAAGTT |
| Subcellular localization | ZmACR10-F | ZmACR10 | ATGAACGCTGCGATCGCCTC |
|  | ZmACR10-R |  | CAGCGGGAGGGAAAACCCGC |
|  | ZmACR20-F | ZmACR20 | ATGCTGCCCTACTTTGATCC |
|  | ZmACR20-R |  | GGCCCGGAGCTTCGCGAAGAG |
|  | ZmACR13-F | ZmACR13 | ATGGAGTGGCTGGACGAGTAC |
|  | ZmACR13-R |  | CGAGCACGACTTGATGAGGC |
|  | ZmACR23-F | ZmACR23 | ATGCCGGTGGGGGAGTTGGC |
|  | ZmACR23-R |  | GTTGTAGCATCCCATCAGCG |

|  | Primer name | Gene name | Primer sequence（5’-3’ |
| --- | --- | --- | --- |
| qRT-PCR | ZmActin-F | ZmActin8 | GCATCCATGAGACCACCTACAAC |
|  | ZmActin-R |  | GATGGACCCTCCTATCCAGACAC |
|  | Zm33878-F | ZmACR5 | GGACTTCGACCCGCTACT |
|  | Zm33878-R |  | CGGACGTGATGGAGGACA |
|  | Zm33167-F | ZmACR4 | ACGATGGCGACTACGAGC |
|  | Zm33167-R |  | GGATGACTCGCAACCTCT |
|  | Zm49660-F | ZmACR13 | GAGGTGTTCGCCGTGCTG |
|  | Zm49660-R |  | GGTCACCACGGAGTAGCC |
|  | Zm06844-F | ZmACR9 | GGTTGTGGGTGACTATGT |
|  | Zm06844-R |  | AAATCACCTTTGTATCCC |
|  | Zm50394-F | ZmACR16 | ATGGTGTCCGGCCTTCTG |
|  | Zm50394-R |  | GGCGAGGTGGCTGTTGAT |
|  | Zm07288-F | ZmACR10 | ATAATGGGTTTGGTTCGC |
|  | Zm07288-R |  | GCTATCTTGCCAGTTCTA |
|  | Zm11051-F | ZmACR26 | AGGACGATGAAGGACTGC |
|  | Zm11051-R |  | TTCTTCCCGTCTACTTGC |
|  | Zm16811-F | ZmACR20 | TCGTCCGAAGCCAACTAC |
|  | Zm16811-R |  | CTCGGATACTCTTCTGGA |
| Overexpression in *Arabidopsis* | OE-F | ZmACR5 | TCGAGCTTTCGCGAGCTCGGTACCGGTACCATGAAGTACGTCTCCGGGCC |
|  | OE-R |  | GCATGCCTGCAGGTCGACTCTAGATCTAGATCAAGACCTGATGTTGTTGAAGTT |
| Subcellular localization | ZmACR10-F | ZmACR10 | ATGAACGCTGCGATCGCCTC |
|  | ZmACR10-R |  | CAGCGGGAGGGAAAACCCGC |
|  | ZmACR20-F | ZmACR20 | ATGCTGCCCTACTTTGATCC |
|  | ZmACR20-R |  | GGCCCGGAGCTTCGCGAAGAG |
|  | ZmACR13-F | ZmACR13 | ATGGAGTGGCTGGACGAGTAC |
|  | ZmACR13-R |  | CGAGCACGACTTGATGAGGC |
|  | ZmACR23-F | ZmACR23 | ATGCCGGTGGGGGAGTTGGC |
|  | ZmACR23-R |  | GTTGTAGCATCCCATCAGCG |

| Table S2. Protein characteristic information of *ZmACR* family genes in maize | | | | | | |
| --- | --- | --- | --- | --- | --- | --- |
| Gene ID | Gene name | chr | position | AA length | MW(Da) | PI |
| *Zm00001d028416* | *ZmACR1* | 1 | 34211830 ~ 34214257 | 440 | 48231.1 | 6.63 |
| *Zm00001d029515* | *ZmACR2* | 1 | 74095473 ~ 74102001 | 489 | 54446 | 6.85 |
| *Zm00001d031639* | *ZmACR3* | 1 | 197105593 ~ 197108840 | 453 | 49713.7 | 5.37 |
| *Zm00001d033167* | *ZmACR4* | 1 | 252662518 ~ 252665880 | 442 | 49836 | 7.84 |
| *Zm00001d033878* | *ZmACR5* | 1 | 277214464 ~ 277217242 | 493 | 54161.8 | 7.06 |
| *Zm00001d003931* | *ZmACR6* | 2 | 69777965 ~ 69780202 | 451 | 49243.7 | 4.96 |
| *Zm00001d005535* | *ZmACR7* | 2 | 177655850 ~ 177675449 | 364 | 38694.1 | 7.7 |
| *Zm00001d006267* | *ZmACR8* | 2 | 203274963 ~ 203284258 | 593 | 65651.2 | 6.16 |
| *Zm00001d006844* | *ZmACR9* | 2 | 217764077 ~ 217767405 | 230 | 25471.4 | 4.33 |
| *Zm00001d007288* | *ZmACR10* | 2 | 226875748 ~ 226883012 | 428 | 46498.9 | 6.8 |
| *Zm00001d040706* | *ZmACR11* | 3 | 59442086 ~ 59443660 | 105 | 11513.8 | 3.92 |
| *Zm00001d041918* | *ZmACR12* | 3 | 143615485 ~ 143625037 | 525 | 57638.2 | 4.91 |
| *Zm00001d049660* | *ZmACR13* | 4 | 39000226 ~ 39002487 | 432 | 46737.7 | 6.35 |
| *Zm00001d049851* | *ZmACR14* | 4 | 47821122 ~ 47823816 | 505 | 55258.6 | 5.4 |
| *Zm00001d050134* | *ZmACR15* | 4 | 68237414 ~ 68255070 | 620 | 67475 | 8.41 |
| *Zm00001d050394* | *ZmACR16* | 4 | 85655816 ~ 85658613 | 370 | 37807.5 | 5.4 |
| *Zm00001d051043* | *ZmACR17* | 4 | 138316944 ~ 138322251 | 483 | 52301.6 | 9.04 |
| *Zm00001d053168* | *ZmACR18* | 4 | 217742350 ~ 217753101 | 566 | 62239.4 | 4.76 |
| *Zm00001d015032* | *ZmACR19* | 5 | 72787738 ~ 72794488 | 569 | 63671.6 | 6.55 |
| *Zm00001d016811* | *ZmACR20* | 5 | 176930372 ~ 176935936 | 460 | 49892 | 6.41 |
| *Zm00001d017168* | *ZmACR21* | 5 | 187805177 ~ 187809851 | 318 | 33954.7 | 9.22 |
| *Zm00001d035119* | *ZmACR22* | 6 | 6131054 ~ 6133096 | 443 | 47564.7 | 6.44 |
| *Zm00001d035634* | *ZmACR23* | 6 | 37855733 ~ 37858713 | 443 | 48938 | 7.32 |
| *Zm00001d021336* | *ZmACR24* | 7 | 149167346 ~ 149179144 | 568 | 63812.1 | 6.47 |
| *Zm00001d021473* | *ZmACR25* | 7 | 153177327 ~ 153198773 | 594 | 65835.6 | 5.87 |
| *Zm00001d011051* | *ZmACR26* | 8 | 137414912 ~ 137417939 | 441 | 48562.2 | 6.69 |
| *Zm00001d023510* | *ZmACR27* | 10 | 8017877 ~ 8030260 | 555 | 61075.2 | 5.17 |
| *Zm00001d026677* | *ZmACR28* | 10 | 149751381 ~ 149758192 | 562 | 62568.8 | 5.58 |

Table S3. Statistics of the Ka/Ks analysis.

| Gene pairs | Method | KA | KS | KA/KS | P-Value (Fisher) |
| --- | --- | --- | --- | --- | --- |
| *ZmACR25* & *ZmACR8* | MA | 0.019 | 0.12 | 0.15 | 2.76E-16 |
| *ZmACR26* & *ZmACR23* | MA | 0.051 | 0.67 | 0.077 | 1.95E-60 |

Table S4 The secondary structure statistics of the selected ZmACR proteins.

|  | Alpha helix | Extended strand | Beta turn | Random coil |
| --- | --- | --- | --- | --- |
| ZmACR26 | 32.81% | 20.73% | 5.77% | 40.68% |
| ZmACR27 | 39.1% | 14.05% | 6.85% | 40% |
| ZmACR17 | 34.99% | 23.19% | 8.7% | 33.1% |
| ZmACR21 | 32.08% | 20.75% | 9.12% | 38.05% |
| ZmACR3 | 38.19% | 18.54% | 6.18% | 37.09% |
| ZmACR5 | 36.11% | 17.85% | 7.1% | 38.95% |
| ZmACR15 | 42.9% | 17.9% | 6.29% | 32.9% |
| ZmACR22 | 39.5% | 18.51% | 6.09% | 35.89% |

Table S5. Detailed information of cis-acting regulation elements of *ZmACR* promoters

| *Cis*-acting elements | Sequence | Potential function | |
| --- | --- | --- | --- |
| A-box | CCGTCC | *Cis*-regulating element | |
| ABRE | ACGTG | *Cis*-acting elements involved in abscisic acid reactions | |
| ACE | GACACGTATG | *Cis*-acting elements involved in light reactions | |
| AE-box | AGAAACAA | Part of the light reaction module | |
| ARE | AAACCA | *Cis*-regulatory elements necessary for anaerobic induction | |
| ATC-motif | AGTAATCT | Part of a conserved DNA module involved in light reactions | |
| ATCT-motif | AATCTAATCC | Part of a conserved DNA module involved in light reactions | |
| AT~TATA-box | TATATA | / | |
| AuxRR-core | GGTCCAT | *Cis*-regulatory element involved in auxin response | |
| Box4 | ATTAAT | Part of a conserved DNA module involved in light reactions | |
| CAAT-box | CAAAT | A common *cis*-acting element of promoter and enhancer regions | |
| CAG-motif | GAAAGGCAGAC | | Part of a photoresponsive element |
| CAT-box | GCCACT | | *Cis*-regulatory elements associated with meristem expression |
| CCAAT-box | CAACGG | | MYBHv1 binding site |
| CGTCA-motif | CGTCA | | *Cis*-regulatory element involved in jasmonic acid reaction |
| DRE | ATTTTAAA | | / |
| E2Fb | TTTGCCGC | | / |
| ERE | ATTTTAAA | | Ethylene responsive element |
| G-Box | CACGTT | | *Cis*-regulatory element involved in light response |
| G-box | TAAACGTG | | *Cis*-regulatory element involved in light response |
| GA-motif | ATAGATAA | | Part of a photoresponsive element |
| GARE-motif | TCTGTTG | | Gibberellin response element |
| GATA-motif | AAGGATAAGG | | Part of a photoresponsive element |
| GC-motif | CCCCCG | | Enhancer like elements associated with specific induction of hypoxia |
| GCN4_motif | TGAGTCA | | *Cis*-regulatory elements involved in endosperm expression |
| GT1-motif | GGTTAA | | Photoresponsive element |
| GTGGC-motif | GATTCTGTGGC | | Part of a photoresponsive element |
| I-box | GTATAAGGCC | | Part of a photoresponsive element |
| LAMP-element | CTTTATCA | | Part of a photoresponsive element |
| LTR | CCGAAA | | *Cis*-acting elements involved in low temperature reactions |
| MBS | CAACTG | | MYB binding sites associated with drought induction |
| MBSI | AAAAAAC（G/C）GTTA | | MYB binding sites involved in flavonoid biosynthesis gene regulation |
| MRE | AACCTAA | | MYB binding sites involved in light response |
| MSA-like | TCCAACGGT | | *Cis*-acting elements involved in cell cycle regulation |
| MYB | TAACCA/CAACAG | | / |
| MYC | CATTTG | | / |
| O2-site | GATGATGTGG | | *Cis*-regulatory elements involved in the regulation of zein metabolism |
| OCT | CGCGGATC | | / |
| P-box | CCTTTTG | | Gibberellin response element |
| Pc-CMA2c | GCCCACGCA | | Part of a photoresponsive element |
| RY-element | CATGCATG | | / |
| STRE | AGGGG | | / |
| Sp1 | GGGCGG | | Photoresponsive element |
| TATA-box | TATA | | The core promoter element |
| TATC-box | TATCCCA | | *Cis*-acting elements associated with gibberellin reactions |
| TCA-element | TCAGAAGAGG | | *Cis*-acting elements related to salicylic acid reactions |
| TCCC-motif | TCTCCCT | | Part of a photoresponsive element |
| TCT-motif | TCTTAC | | Part of a photoresponsive element |
| TGA-box | TGACGTAA | | Part of an auxin reaction element |
| TGA-element | AACGAC | | Auxin reaction element |
| TGACG-motif | TGACG | | *Cis*-regulatory element involved in jasmonic acid reaction |
| W box | TTGACC | | Injury and pathogen response elements |
| WRE3 | CCACCT | | / |
| WUN-motif | AAATTACT | | / |
| as-1 | TGACG | | / |
| chs-CMA2a | TCACTTGA | | Part of a photoresponsive element |
| circadian | CAAAGATATC | | *Cis*-regulatory elements involved in circadian rhythm control |
| dOCT | CTCGGATC | | / |
| plant_AP-2-like | CGACCAGG | | / |
| re2f-1 | GCGGGAAA | | / |
